# Chromosome-level genome assembly reveals the unique genome evolution of the swimming crab (*Portunus trituberculatus*)

**DOI:** 10.1093/gigascience/giz161

**Published:** 2020-01-06

**Authors:** Boping Tang, Daizhen Zhang, Haorong Li, Senhao Jiang, Huabin Zhang, Fujun Xuan, Baoming Ge, Zhengfei Wang, Yu Liu, Zhongli Sha, Yongxu Cheng, Wei Jiang, Hui Jiang, Zhongkai Wang, Kun Wang, Chaofeng Li, Yue Sun, Shusheng She, Qiang Qiu, Wen Wang, Xinzheng Li, Yongxin Li, Qiuning Liu, Yandong Ren

**Affiliations:** 1 Jiangsu Key Laboratory for Bioresources of Saline Soils, Jiangsu Provincial Key Laboratory of Coastal Wetland Bioresources and Environmental Protection, Jiangsu Synthetic Innovation Center for Coastal Bio-agriculture, Yancheng Teachers University, Kaifang Road 50#, Yancheng 224002, China; 2 Center for Ecological and Environmental Sciences, Northwestern Polytechnical University, Youyixi Road 127#, Xi'an 710072, China; 3 Institute of Oceanology, Chinese Academy of Sciences, Nanhai Road 7#, Qingdao 266071, China; 4 Key Laboratory of Freshwater Aquatic Genetic Resources, Ministry of Agriculture and Rural Affairs, Shanghai Ocean University, Huchenghuan Road 999#, Shanghai 201306, China; 5 National Engineering Laboratory of Marine Germplasm Resources Exploration and Utilization, Zhejiang Ocean University, Haidanan Road 1#, Zhoushan 316022, China; 6 National Engineering Research Center for Facilitated Marine Aquaculture, Zhejiang Ocean University, Haidanan Road 1#, Zhoushan 316022, China; 7 China Hong Kong Ecology Consultant Company, Lam Tsuen, Chai Kek, 96#, Hong Kong, China

**Keywords:** *Portunus trituberculatus*, genome assembly, crab, chromosome, evolution

## Abstract

**Background:**

The swimming crab, *Portunus trituberculatus*, is an important commercial species in China and is widely distributed in the coastal waters of Asia-Pacific countries. Despite increasing interest in swimming crab research, a high-quality chromosome-level genome is still lacking.

**Findings:**

Here, we assembled the first chromosome-level reference genome of *P. trituberculatus* by combining the short reads, Nanopore long reads, and Hi-C data. The genome assembly size was 1.00 Gb with a contig N50 length of 4.12 Mb. In addition, BUSCO assessment indicated that 94.7% of core eukaryotic genes were present in the genome assembly. Approximately 54.52% of the genome was identified as repetitive sequences, with a total of 16,796 annotated protein-coding genes. In addition, we anchored contigs into chromosomes and identified 50 chromosomes with an N50 length of 21.80 Mb by Hi-C technology.

**Conclusions:**

We anticipate that this chromosome-level assembly of the *P. trituberculatus* genome will not only promote study of basic development and evolution but also provide important resources for swimming crab reproduction.

## Introduction

The swimming crab, *Portunus trituberculatus* (NCBI:txid210409, marinespecies.org:taxname:1061762), belonging to Brachyura, Portunidae, Portunus, is named for its shuttle-shaped head breastplate and 3 verrucous bumps on the back of the stomach and heart regions [1, 2]. The chelipeds of swimming crabs are well developed for feeding and attacking, with the first 3 pairs and last pair used for crawling and swimming, respectively [3, 4]. Male and female crabs are distinguished by the shape of their abdomen, with the male having a triangular abdomen and the female having an almost circular one [5]. Owing to their lack of drilling ability, swimming crabs often live in soft mud or sand [6] or in seagrass near the shore, and also show a certain level of phototaxis, spending time on the sea floor during the day and foraging at night [5]. Swimming crabs are also omnivorous, feeding on shellfish, small fish, shrimp, algae, and decomposing animal and plant carcasses [7].

The swimming crab is widely distributed in the coastal waters of Korea, Japan, China, and Southeast Asia and is one of the most valuable marine crustaceans in Asia [8]. It is widely found in Chinese coastal waters of the Bohai Sea, Yellow Sea, East China Sea, and South China Sea and is an important commercially cultured species [9]. Swimming crabs are considered highly nutritious, especially in regard to crab cream, and are very popular in China [10, 11]. As a result, the crab has been heavily overfished, resulting in substantial declines in its natural population [12] and initiation of artificial breeding [13, 14]. With continued research on the crab, its morphological and physiological characteristics have become clear, but the genetic changes are poorly understood. At present, several genomic studies of swimming crab have been carried out [15–18], but the high-quality chromosome-level genome is still lacking.

In the present study, we constructed a chromosome-level genome assembly of *P. trituberculatus* by combining short reads, Nanopore long reads, and Hi-C sequencing data. This chromosome-level genome will not only promote study on development and evolution but also provide important resources for reproductive studies of *P. trituberculatus* and other crab species.

## Methods

### Sampling, library construction, and sequencing

**Figure 1:**
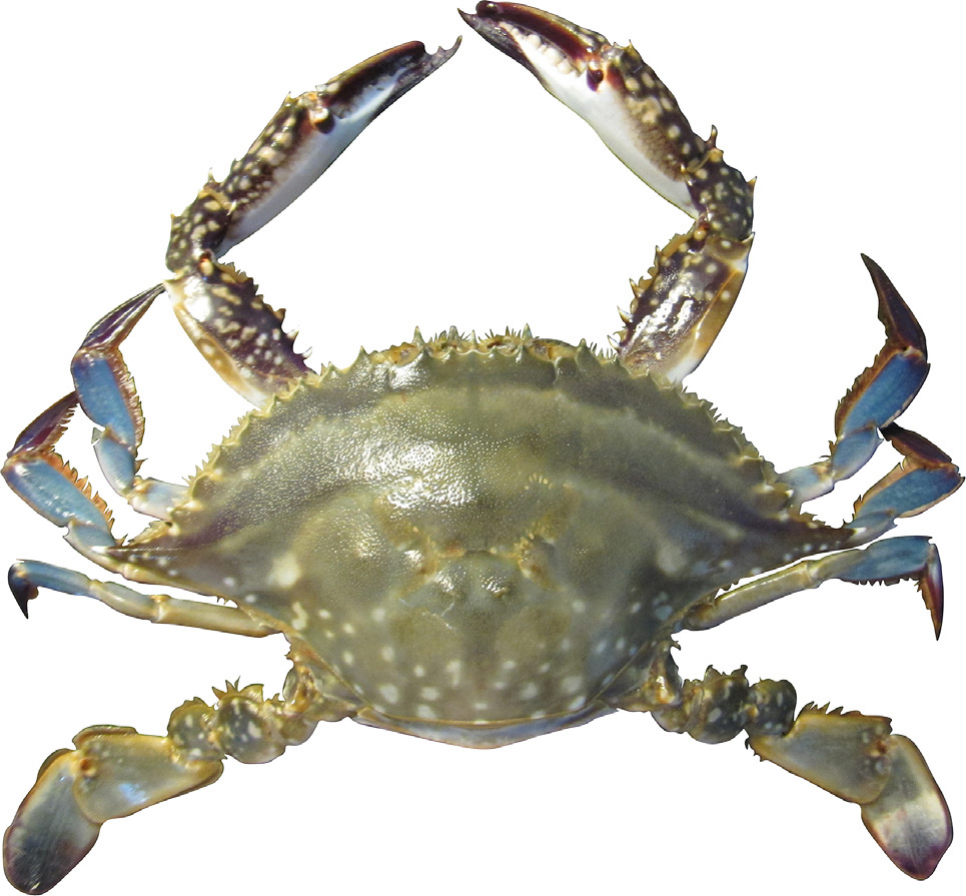
Swimming crab, *Portunus trituberculatus*. The adult male swimming crab collected from Bohai Bay, Hebei Province.

A male swimming crab was collected in Bohai Bay, Hebei Province, China, for sequencing (Fig. 1). To obtain sufficient high-quality DNA for the Oxford Nanopore (Oxford, UK) and BGISEQ-500 platforms (BGI, Qingdao, China), the swimming crab was rinsed 5 times with clean water and dissected immediately. Fresh muscle tissue was collected and snap-frozen in liquid nitrogen. The samples were then used to extract DNA with a Qiagen Blood & Cell Culture DNA Mini Kit and prepared for Nanopore, BGISEQ-500, and Hi-C sequencing. Using the same individual, muscle RNA was also extracted using TRIzol (Invitrogen) according to the manufacturer's instructions. To obtain an overview of the transcriptome, polyadenylated RNA was chosen by oligo (dT) purification and reverse-transcribed to complementary DNA and sequenced using the BGISEQ-500 platform.

Extracted DNA was sequenced using both the BGISEQ and Oxford Nanopore platforms. The short reads generated from the BGISEQ platform were used for estimation of genome size and error correction of the assembled genome, and the Nanopore long reads were used for genome assembly. To this end, 1 library with insertion lengths of ∼300 bp was sequenced on the BGISEQ-500 platform, and another library with an average length of 20 kb was constructed using the Oxford Nanopore platform according to the manufacturers’ protocols.

### Data filtering

Three different sources of reads were used to achieve the high-quality genome assembly, i.e., Nanopore long reads, short reads, and Hi-C reads. Thus, we used different methods for filtering. For the Nanopore long reads, any reads <1 kb or with a mean quality value of <7 were removed. For the short reads, any read with >10% unknown bases (usually designated “N”) or with >50% low-quality bases was removed, and its paired-end read was also removed. All adaptor sequences and duplicated reads produced by PCR were removed. The low-quality Hi-C reads were filtered using HiC-Pro v2.10.0 [19] with default parameters.

### Genome characteristic estimation

All filtered BGISEQ short reads were used for estimation of genome size and other characteristics. In addition, 17-mer was chosen for *k*-mer analysis and the 17-mer depth frequency distribution was calculated using the *k*-mer method. Genome size was estimated as follows: genome size = TKN17-mer/PKFD17-mer, where TKN17-mer is the total *k*-mer number and PKFD17-mer is the peak *k*-mer frequency depth of 17-mer. The estimated genome size was used to determine subsequent genome assembly results.

### Genome assembly

To improve the quality of the genome and reduce the error ratio, self-error correction of all Nanopore long reads was performed using NextDenovo software [20]. The error-corrected Nanopore long reads were then used to assemble the raw genome via contig construction with WTDBG software (WTDBG, RRID:SCR_017225) [21] and the following parameters: -p 0 -k 15 -AS 2 -E 1 -s 0.05 -L 5000. The assembled genomic sequences were further polished by Racon v1.2.1 [22] with 4 iterations using all the error-corrected Nanopore long reads with default parameters. After this, all filtered BGISEQ short reads were polished by Pilon v1.21 (Pilon, RRID:SCR_014731) [23] at the single-base level with default parameters. After completion of the error correction steps, the Hi-C data were used to obtain a chromosome-level genome assembly. All Hi-C sequencing data were first filtered by HiC-Pro v2.10.0 [19] with default parameters and then mapped to the polished swimming crab genome to improve the connection integrity of the contigs. Finally, 3D *de novo* assembly software (v180419) [24] with default parameters was used to determine contig location and direction.

### Genome assembly evaluation

Three different strategies were used to evaluate the completeness and accuracy of the assembled genome. First, the quality of the assembled genome and gene completeness were assessed using BUSCO (BUSCO, RRID:SCR_015008) [25] with the core gene sets of the eukaryote and metazoan databases, respectively. Second, all filtered short reads generated by BGISEQ were mapped to the assembled genome using BWA-MEM v0.7.12 [26] to detect genome integrity with default parameters. Third, transcripts were mapped to the assembled genome using BLAT software (BLAT, RRID:SCR_011919) [27] with e-value <10−5.

### Repetitive element annotation

Tandem repeats and transposable elements (TEs) were also annotated in the chromosome-level genome. Tandem repeats were annotated using Tandem Repeat Finder v4.04 [28] with default parameters. The TEs were annotated at the protein level using RepeatProteinMask (RM-BLASTX) to search the protein database and at the DNA level using RepeatMasker (open-4.0.7) (RepeatMasker, RRID:SCR_012954) [29] to search the *de novo* libraries and repbase. The *de novo* repeat libraries were constructed using RepeatModeler (RepeatModeler, RRID:SCR_015027) [30], with consensus sequences used for *de novo* library construction, and all software using the default parameters.

### Gene structure prediction and function annotation

After repetitive element annotation, the repeat-masked genome was used for gene set annotation with 3 different methods, i.e., *de novo* prediction, RNA-seq–based annotation, and homology-based annotation. We first assembled the RNA-seq reads into transcripts using Bridger r2014–12-01 (Bridger, RRID:SCR_017039) [31]. The assembled genome and transcripts were then used for Augustus training to obtain an accurate Augustus annotation species model. Augustus v2.5.5 (Augustus, RRID:SCR_008417) [32] was used for *de novo* prediction of coding genes with the previous training results. Second, proteins of *Bicyclus anynana* (GCF_900239965.1) [33], *Bombus terrestris* (GCF_000214255.1) [34], *Drosophila melanogaster* (GCA_000001215.4) [35], *Mus musculus* (GCF_000001635.26) [36], *Stegodyphus mimosarum* (GCA_000611955.2), *Penaeus vannamei* (GCA_003789085.1), *Mesobuthus martensii* [37], *Eriocheir japonica sinensis* (i.e., *Eriocheir sinensis*) (GigaDB: 100186) [38–43], and *Tachypleus tridentatus* (GCA_004102145.1) [44] were downloaded from the NCBI, GigaDB, or their own databases. The longest transcript of each gene was selected for further annotation and phylogenetic analysis. All filtered genes were searched with an e-value cutoff of 1e−5, with the blast results then formatted and prepared for Genewise [45] prediction of the gene structure of the swimming crab genome. Third, for the RNA-seq–based method, all assembled transcripts were aligned against the genome using BLAT [27] (identity > 90% and coverage > 90%), with PASA used to filter overlaps to link the spliced alignments. Finally, EvidenceModeler (EVM; EVidenceModeler, RRID:SCR_014659) v1.1.1 was used to integrate the above data into an EVM-derived gene set [46].

Five different public protein databases were used for gene functional annotation of the swimming crab, with InterProScan v4.8 (InterProScan, RRID:SCR_005829) [47] used to screen proteins against the 5 databases (Pfam, release 27.0; PRINTS, release 42.0; PROSITE, release 20.97; ProDom, 2006.1; and SMART, release 6.2) to determine the number of InterPro- and GO-predicted protein-coding genes. In addition, the KEGG, UniProt/SwissProt, and UniProt/TrEMBL databases were also used for functional annotation with BLAST v2.3.0 [48]. Blastp (BLASTP, RRID:SCR_001010) was used in this step, and the e-value was set as 10−5 and other parameters were set as defaults.

### Identification of orthologous genes

The annotated genes in the swimming crab and 6 other species, including *Aedes aegypti* (GCF_002204515.2), *B. anynana, D. melanogaster, S. mimosarum, P. vannamei*, and *E. j. sinensis*, were used for orthologous gene identification with OrthoMCL v2.0.9 [49] with default parameters. The identified genes were then used to run reciprocal alignment and pairwise relationship analysis. The reciprocal best similarity pairs in different species were considered as putative orthologous genes, and reciprocal better similarity pairs in 1 species were considered as paralogous genes. The 1:1:1:1:1:1:1 single-copy genes in the 7 species were also identified for further phylogenetic and divergence time estimation analysis.

### Phylogenetic analysis and divergence time estimation

Using the single-copy genes of the 7 species (*P. trituberculatus, A. aegypti, B. anynana, D. melanogaster, S. mimosarum, P. vannamei*, and *E. j. sinensis*), we connected the genes in each species into 1 super-gene for phylogenetic tree building. Maximum likelihood–based phylogenetic analysis was conducted using RAxML v8.2.10 (RAxML, RRID:SCR_006086) [50] with default parameters. The MCMCTREE program in the PAML package v4.8 [51] was then used to calculate divergence time, with all fossil records downloaded from the TIMETREE website [52] for calibration.

### Relative evolution rate

The relative evolution rate of species was analyzed with LINTRE software (version 1) [53] using the "tpcv" model and *S. mimosarum* as an outgroup. Using the default parameters of LINTRE, we then evaluated the relative evolution rate between the swimming crab and other related species.

### Gene family expansion and contraction

Using the divergence time results calculated by MCMCTREE and the gene pairwise relationships calculated by OrthoMCL [49], we determined gene family expansion and contraction for each node using CAFÉ v3.1 (CAFÉ, RRID:SCR_005983) [54]. The expansion and contraction genes of the swimming crab were extracted for GO/KEGG enrichment analysis [55, 56].

## Results

### Chromosome-level genome assembly

**Figure 2:**
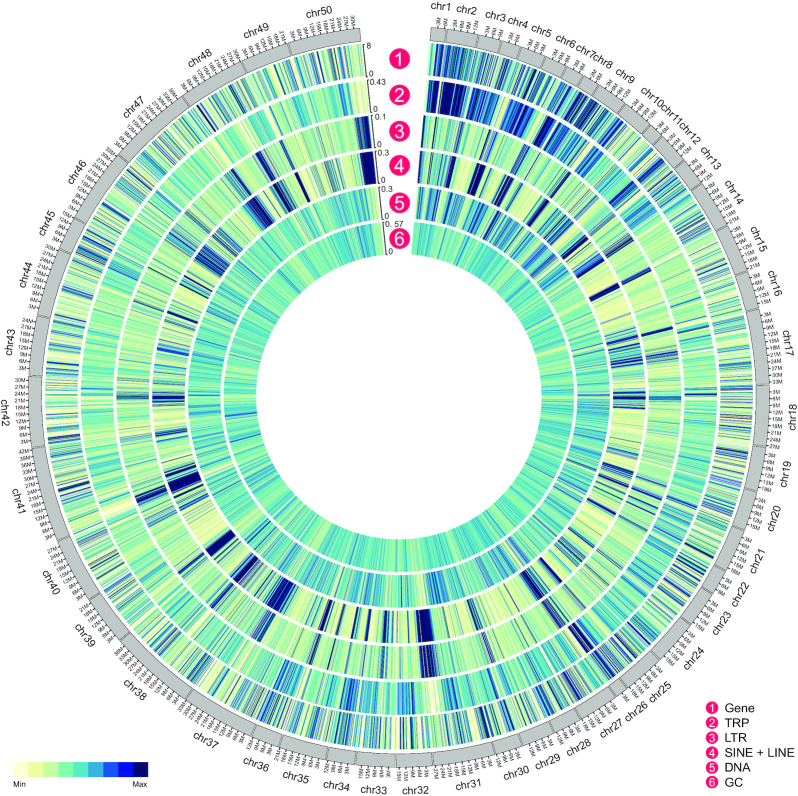
Genome characteristics of swimming crab. From outer circle to inner circle: gene distribution, tandem repeats (TRP), long tandem repeats (LTR), long interspersed nuclear elements (LINE) and short interspersed nuclear elements (SINE), the DNA elements, and the GC content of the genome.

To obtain a high-quality chromosome-level swimming crab genome, we extracted high-quality DNA from the muscle tissue and constructed libraries for genome sequencing. To estimate the genome characteristics of the swimming crab, we generated 205.40 Gb of BGISEQ data (Table S1), with 17-mer analysis indicating a genome size of ∼918.52 Mb and a heterozygosity rate of ∼0.9% (Fig. S1). In total, we generated 54.97 Gb (54.75-fold coverage) of Nanopore long-read data with N50 >20 kb (Table S2). The Nanopore long reads were assembled into contigs using WTDBG software [21] (genome size: 1.00 Gb; N50: 4.12 Mb) (Table 1). To further improve genome accuracy, we aligned all corrected Nanopore long reads to the assembled genome and conducted error correction using Racon [22] with 4 iterations. The genome was subsequently corrected using all filtered BGISEQ clean reads via Pilon [23] with 2 iterations. We then constructed the chromosome-level genome with 95.95 Gb of Hi-C sequencing data (Table S3) by 3D *de novo* assembly [24]. Finally, we obtained 50 chromosomes and a mounting rate (total length of the contigs that anchored to chromosomes divided by the total length of all assembled contigs) of 97.80% (Fig. 2; Table S4), which is the first chromosome-level crab genome with N50 of 21.79 Mb (Table 1). The high mounting rate suggested successful assembly of the swimming crab genome at the chromosome level. We also compared our assembled genome to the published swimming crab genome; the assembly quality of our genome is better than the previous one (Table S5). Because the previous study has the genomic markers, we also mapped all the markers to our genome, and we found that 99.40% (10,897 of 10,963) markers can be mapped to our genome. Among these mapped genome markers, 98.83% (10,769 of 10,897) are exactly mapped to our assembled 50 chromosomes (Table S6). All these results show that we obtained a high-quality and quite complete chromosome-level genome.

**Table 1:** Assembly of swimming crab genome

Term	Contig phase		Hi-C phase	
	Size (bp)	Number	Size (bp)	Number
N90	439,683	334	11,273,125	41
N80	1,225,551	203	14,151,211	33
N70	2,035,154	141	16,942,622	27
N60	2,950,146	100	19,786,189	21
N50	4,121,416	71	21,793,880	17
Maximum length	17,984,318	-	42,710,960	-
Total length	1,004,084,521	-	1 005,046,021	-
No. ≥100 bp	-	2,446	-	523
No. ≥10 kb	-	1,756	-	314

Note: Contig phase represents results assembled by WTDBG software, and Hi-C phase represents scaffold statistics of genome after chromosome assembly.

### Genome quality evaluation

We next assessed the completeness of the swimming crab genome by BUSCO [25] and identified 94.7% Eukaryota and 92.9% Metazoa conserved core genes in the genome (Table 2). We checked the mapping rates of the BGISEQ short reads to our genome and found that 95.85% of reads were properly pair-mapped to the genome (Table S7). We then *de novo* assembled the transcripts using the RNA-seq data (Table S8) with Bridger software [31] and an N50 length of 2,124 bp (Table S9). After transcript mapping, we found that 97.80% of the transcripts could be mapped to the swimming crab genome (Table S10). We also analyzed the genome quality of previously published high-quality genomes from closely related species and determined that the quality of the assembled chromosome-level swimming crab genome was markedly higher or comparable to that of other species (Table S11). In summary, these results indicated that we acquired a high-quality swimming crab genome. To investigate genome characteristics, such as GC content, we analyzed the GC distribution in the genome with a slide-window method. The peak value of GC content was ∼41%, which agrees with the average GC content in the swimming crab genome. We also found that the GC content in the swimming crab was closer to that of mouse than of shrimp (Fig. S2).

**Table 2:** Quality evaluation of assembled swimming crab genome by BUSCO

Library	Eukaryota	Metazoa
Complete BUSCO (C)	287	909
Complete and single-copy BUSCO (S)	283	903
Complete and duplicated BUSCO (D)	4	6
Fragmented BUSCO (F)	2	19
Missing BUSCO (M)	14	50
Total BUSCO groups searched	303	978
Percentage of complete BUSCO (%)	94.7	92.9

### Genome annotation

The repetitive sequences of the swimming crab genome were identified through 4 different methods, resulting in 547.39 Mb of repeated sequences and accounting for 54.52% of the assembled genome (Table S12). Among the repeated sequences, 19.28% (∼193.56 Mb) were tandem repeats and 52.29% (∼525.49 Mb) were TEs (Table S12; Table 3). The TEs could be further divided into 4 main types, including 0.014% (∼142.88 kb) of short interspersed nuclear elements (SINEs), 15.23% (∼153.03 Mb) of long interspersed nuclear elements (LINEs), 14.90% (∼149.71 Mb) of DNA elements, and 4.50% (∼45.19 Mb) of long terminal repeats (LTRs) (Table 3).

**Table 3:** Statistics on transposable elements in swimming crab genome

Type	Repbase TEs		TE proteins		*De novo*		Combined TEs	
	Length (bp)	% in genome	Length (bp)	% in genome	Length (bp)	% in genome	Length (bp)	% in genome
DNA	131,799,733	13.11	2,434,533	0.24	19,288,080	1.92	149,711,951	14.90
LINE	16,171,649	1.61	75,759,827	7.54	131,530,457	13.09	153,027,744	15.23
SINE	142,878	0.01	0	0	0	0	142,878	0.014
LTR	26,546,055	2.64	10,195,324	1.01	18,421,957	1.83	45,189,365	4.50
Other	89,969,319	8.95	0	0	211,157,523	21.01	230,116,216	22.90
Unknown	34,752	0.0035	0	0	90,989,908	9.05	91,007,921	9.06
Total	213,558,503	21.25	88,375,336	8.79	464,908,824	46.26	525,492,271	52.29

**Figure 3:**
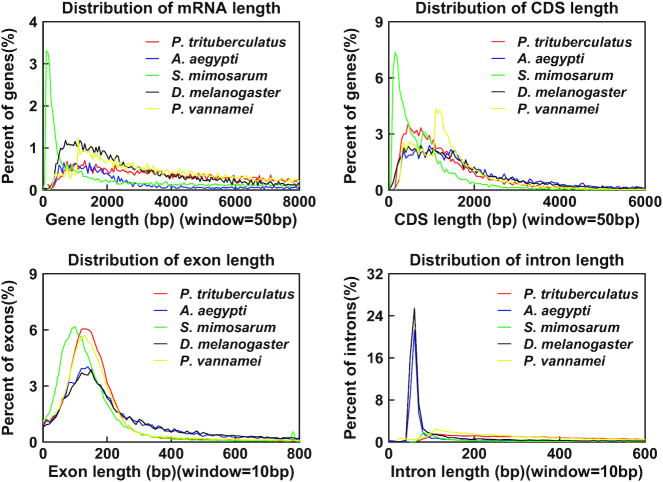
Annotation quality comparison of protein-coding genes. We compared the messenger RNA (mRNA) length, CDS length, exon length, and intron length among 5 species: *P. trituberculatus, A. aegypti, S. mimosarum, D. melanogaster*, and *P. vannamei*.

After masking the repeated sequences, we annotated the protein-coding genes using *de novo* prediction, homology-based prediction, and transcript-based prediction. We merged the results and obtained 16,791 protein-coding genes. We checked the quality of the annotated genes by comparing with several closely related species. Results showed that the messenger RNA, CDS, exon, and intron length distributions of the swimming crab were similar to those of the closely related species, suggesting that the swimming crab annotation results were dependable (Fig. 3).

We also performed functional annotation of the 16,791 genes with InterPro, GO, KEGG, SwissProt, and TrEMBL. The highest annotation rate (74.77%) was found for SwissProt, in which 12,558 genes were annotated. In total, 16,053 genes (∼95.58%) were annotated, indicating that most genes could be found in the public protein databases (Table 4). Thus, taken together, we acquired a high-quality protein-coding gene set for the swimming crab.

**Table 4:** Functional annotation of predicted protein-coding genes

Term	Gene number	Percentage (%)
GO	8,712	51.87
InterPro	11,691	69.61
KEGG	10,880	64.78
SwissProt	12,558	74.77
TrEMBL	12,256	72.97
Annotated	16,053	95.58
Unannotated	743	4.42
Total	16,796	100

### Orthologous identification and gene family analysis

**Figure 4:**
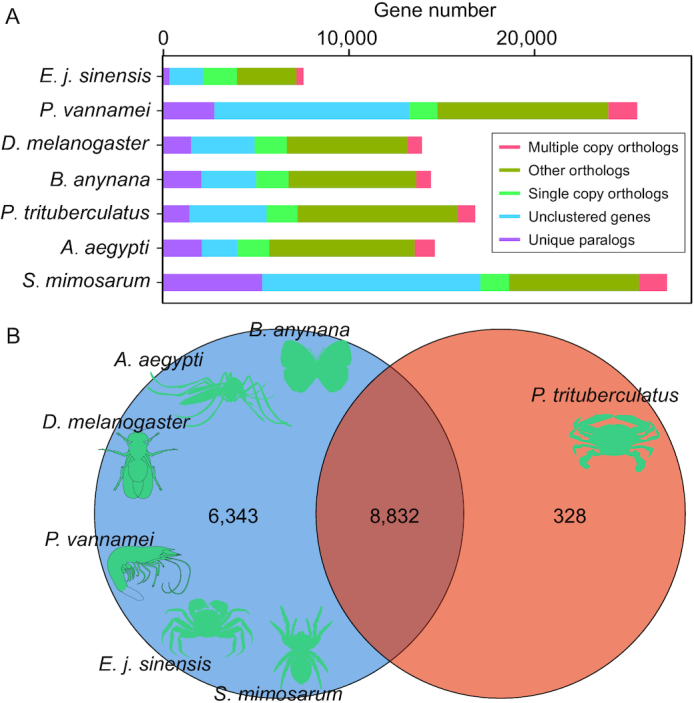
Gene family analysis of swimming crab. A. Orthologous genes among species. The multiple-copy orthologs are orthologs that have multiple copies in 1 species, the single-copy orthologs are orthologs that have only 1 copy in 1 species, the other orthologs are the rest of the orthologs, the unclustered genes are genes that have no homology with others, and the unique paralogs are genes that only exist in 1 specific species. B. Unique and common gene families among these species, including *B. anynana, A. aegypti, D. melanogaster, P. vannamei, E. j. sinensis, S. mimosarum*, and *P. trituberculatus*.

For comparative genomics analysis of the swimming crab, we analyzed the orthologous gene relationships among several species, including *A. aegypti, B. anynana, D. melanogaster, S. mimosarum, P. vannamei*, and *E. j. sinensis* using OrthoMCL. In total, 15,503 gene families were clustered in the 7 species and 1,018 one-to-one single-copy genes were identified (Fig. 4A). Because the swimming crab has several unique characteristics, we performed gene family analysis and found 8,832 gene families shared among the 7 species, with 328 gene families unique to the swimming crab (Fig. 4B). We then performed functional analysis and identified 34 enriched KEGG terms (Table S13), suggesting that these unique gene families play important roles in the swimming crab.

### Phylogenetic relationships and divergence time

**Figure 5:**
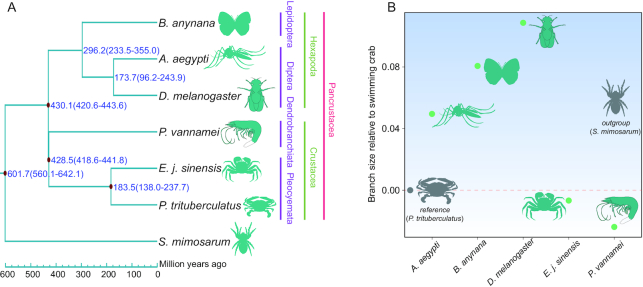
Phylogenetic relationships, divergence time, and evolution rate analysis. A. Phylogenetic relationship and divergence time of species. Red dot represents fossil record used here, and numbers in parentheses indicate 95% confidence interval. B. Relative evolution rate of species.

Although the phylogenetic relationships of the swimming crab and closely related species have been analyzed in previous studies, most used few nuclear and mitochondrial genes. To determine the evolutionary relationship of the swimming crab, we analyzed all single-copy genes using RAxML software [50], with the spider used as the outgroup species. Results showed that the swimming crab has a close relationship with the Chinese mitten crab and shrimp (Fig. 5A). The 7 species of pancrustaceans—*P. trituberculatus, A. aegypti, B. anynana, D. melanogaster, S. mimosarum, P. vannamei*, and *E. j. sinensis—*formed 2 clades: i.e., Hexapoda and Crustacea. The Hexapoda group consisted of all lepidopteran and dipterous insects, whereas the second clade comprised all other crustaceans, with *P. trituberculatus* and *E. j. sinensis* forming a Pleocyemata clade, followed by Dendrobranchiata shrimp (*P. vannamei*). In addition, Hexapoda and Crustacea were both found to be monophyletic (Fig. 5A). To determine divergence time, we employed MCMCTREE analysis in the PAML package [51] and found that the Chinese mitten crab and swimming crab diverged ∼183.5 million years ago, and diverged from shrimp ∼428.5 million years ago (Fig. 5A).

### Relative evolution rate

Species in different environments can experience different survival pressures. As such, we conducted relative evolution rate analysis in LINTRE (version 1) [53], with spider as the outgroup species and swimming crab as the reference species. Results showed that the shrimp had the slowest evolution rate among the 7 species, whereas the fruit fly and butterfly exhibited relatively fast evolution rates (Fig. 5B; Table S14). Interestingly, the slowest evolution rates were found among the Malacostraca (Fig. 5B; Table S14), suggesting that the specific environments or habitats caused their different evolution rates.

### Gene family expansion and contraction

We performed gene family expansion and contraction analysis of the 7 species using CAFÉ v4.0 and identified 148 and 25 expanded and contracted gene families (*P* < 0.05) in the swimming crab, respectively. We then performed KEGG functional enrichment analysis of the expanded gene families and found that the HIF-1 signaling pathway (*Q*-value = 0.000109025), focal adhesion (*Q*-value = 0.000135977), Hippo signaling pathway (*Q*-value = 0.000184649), and insulin signaling pathway (*Q*-value = 0.000357592) were enriched (Table S15). These biological processes are related to early development, hypoxia adaptation, and other key processes, which may help us better understand the evolution of the swimming crab.

## Conclusions

Based on BGISEQ, Nanopore, and Hi-C sequencing data, we assembled a chromosome-level high-quality genome of the swimming crab. Evaluation results indicated that the genome quality of swimming crab was comparable to that of most high-quality model species. We also successfully obtained 16,791 high-quality protein-coding genes by integrating 3 different methods. The genome and annotation data will help researchers better understand the evolution of crabs and improve their economic value. The phylogenetic results indicated that the swimming crab is closely related to the Chinese mitten crab, from which it diverged ∼183.5 million years ago. The unique and/or expanded gene family analysis provides clues to swimming crab development and environmental adaptation.

## Availability of Supporting Data and Materials

The raw sequencing data were deposited in the NCBI database under accession number PRJNA555262. The genome assembly and annotation results are available via the *GigaScience* repository GigaDB [57].

## Additional Files

**Table S1:** Statistics on genome sequencing data from BGISEQ platform.

**Table S2:** Statistics on sequencing reads from Oxford Nanopore platform.

**Table S3:** Statistics on Hi-C sequencing data.

**Table S4:** Statistics on assembled chromosome-level genome by 3D *de novo* assembly software.

**Table S5:** The quality comparison of these 2 genomes.

**Table S6:** The mapping results of genomic markers to the assembled genome.

**Table S7:** Statistics on mapping ratio of the BGISEQ short reads to swimming crab genome.

**Table S8:** Statistics on RNA-seq data.

**Table S9:** Statistics on assembled transcripts by Bridger software.

**Table S10:** Statistics on transcript mapping ratio of swimming crab genome.

**Table S11:** Genome quality comparison of swimming crab with other species.

**Table S12:** Statistics on annotated repetitive sequences using different software.

**Table S13:** KEGG enrichment analysis of unique gene families in swimming crab relative to 6 other species.

**Table S14:** Two-cluster analysis of swimming crab and other species.

**Table S15:** KEGG enrichment analysis of expanded gene families in swimming crab.

**Figure S1:** 17-mer analysis of swimming crab genome.

**Figure S2:** GC distribution in species.

giz161_GIGA-D-19-00309_Original_Submission

giz161_GIGA-D-19-00309_Revision_1

giz161_Response_to_Reviewer_Comments_Original_Submission

giz161_Reviewer_1_Report_Original_Submission Hugues Roest Crollius -- 10/4/2019 Reviewed

giz161_Reviewer_2_Report_Original_Submission Joseph F. Ryan -- 10/4/2019 Reviewed

giz161_Supplemental_Tables_and_Figures

## Abbreviations

BLAST: Basic Local Alignment Search Tool; BLAT: BLAST-Like Alignment Tool; bp: base pairs; BUSCO: Benchmarking Universal Single-Copy Orthologs; BWA: Burrows-Wheeler Aligner; CDS: coding DNA sequence; Gb: gigabase pairs; GC: guanine-cytosine; GO: Gene Ontology; Hi-C: High-throughput chromosome conformation capture; kb: kilobase pairs; KEGG: Kyoto Encyclopedia of Genes and Genomes; Mb: megabase pairs; LINE: long interspersed nuclear element; LTR: long terminal repeat; NCBI: National Center for Biotechnology Information; PASA: Program to Assemble Spliced Alignments; RAxML: Randomized Axelerated Maximum Likelihood; RNA-seq: RNA sequencing; SINE: short interspersed nuclear element; TE: transposable element.

## Competing Interests

The authors declare that they have no competing interests.

## Funding

This study was supported by the National Natural Science Foundation of China (31672267, 31640074), Jiangsu Agriculture Science and Technology Innovation Fund (CX(18)3027, CX(18)2027), Natural Science Foundation of Jiangsu Province (BK20171276, BK20160444), “Qing Lan Project” of Daizhen Zhang and China Postdoctoral Science Foundation (2018M642105).

## Authors' Contributions

Y.R., Q.L., Y. Li, and X.L. conceived the project. B.T., D.Z., S.S., H.Z., Y. Liu, and S.J. collected and dissected the samples. H.L., Zhongkai Wang, K.W., Y.S., Q.Q., C.L., and Y. Li estimated genome size. F.X., Y.C., W.J., and H.J. assembled the genome. B.G., Zhengfei Wang, Z.S., and B.T. performed genome assembly, genome annotation, and evolution analysis. Y.L., B.T., Q.Q., and W.W. wrote the manuscript. Y.R. and W.W. revised the manuscript.
