# Supplementary material for: Chromosome-level genome assembly reveals the unique genome evolution of the swimming crab (Portunus trituberculatus)
Source: Gigascience. 2020 Jan 6;9(1):giz161. doi: 10.1093/gigascience/giz161 (PMC6944217; doi:10.1093/gigascience/giz161)
Supplement: giz161_GIGA-D-19-00309_Revision_1 [file giz161_giga-d-19-00309_revision_1.pdf]

GigaScience

Chromosome-level genome assembly reveals the unique genome evolution of swimming crab (Portunus trituberculatus)

--Manuscript Draft--

Manuscript Number:	GIGA-D-19-00309R1
Full Title:	Chromosome-level genome assembly reveals the unique genome evolution of swimming crab (Portunus trituberculatus)
Article Type:	Data Note
Funding Information:	National Natural Science Foundation of China (31672267) — Dr Boping Tang<br>National Natural Science Foundation of China (31640074) — Mr Qiuning Liu<br>Jiangsu Agricultural Science and Technology Innovation Fund (CX(18)3027) — Dr Boping Tang<br>Jiangsu Agricultural Science and Technology Innovation Fund (CX(18)2027) — Dr Boping Tang<br>Natural Science Foundation of Jiangsu Province (BK20171276) — Dr Daizhen Zhang<br>Natural Science Foundation of Jiangsu Province (BK20160444) — Mr Qiuning Liu<br>Qinglan Project of Jiangsu Province of China (CN) (2018M642105) — Dr Daizhen Zhang
Abstract:	Background: The swimming crab, Portunus trituberculatus, is an important commercial species in China and is widely distributed in the coastal waters of Asia-Pacific countries. Despite increasing interest in swimming crab research, a high quality chromosome-level genome is still missing. Findings: Here, we assembled the first chromosome-level reference genome of P. trituberculatus by combining the short reads, Nanopore long reads, and Hi-C data. The genome assembly size was 1.00 Gb with a contig N50 length of 4.12 Mb. In addition, BUSCO assessment indicated that 94.7% of core eukaryotic genes were present in the genome assembly. Approximately 54.52% of the genome was identified as repetitive sequences, with a total of 16,796 annotated protein-coding genes. In addition, we anchored contigs into chromosomes and identified 50 chromosomes with a N50 length of 21.80 Mb by Hi-C technology. Conclusions: We anticipate that this chromosome-level assembly of the P. trituberculatus genome will not only promote study of basic development and evolution but also provide important resources for swimming crab reproduction.
Corresponding Author:	Yandong Ren<br>Northwestern Polytechnical University<br>Xi'an, Shaanxi CHINA
Corresponding Author Secondary Information:	
Corresponding Author's Institution:	Northwestern Polytechnical University
Corresponding Author's Secondary Institution:	
First Author:	Yandong Ren
First Author Secondary Information:	
Order of Authors:	Yandong Ren<br>Boping Tang

Powered by Editorial Manager® and ProduXion Manager® from Aries Systems Corporation

	Daizhen Zhang
	Haorong Li
	Senhao Jiang
	Fujun Xuan
	Baoming Ge
	Zhengfei Wang
	Yu Liu
	Zhongli Sha
	Yongxu Cheng
	Wei Jiang
	Hui Jiang
	Zhongkai Wang
	Kun Wang
	Chaofeng Li
	Yue Sun
	Shusheng She
	Qiang Qiu
	Wen Wang
	Xinzheng Li
	Yongxin Li
	Qiuning Liu
**Order of Authors Secondary Information:**	
**Response to Reviewers:**	Reviewer #1 Comments for the Author…<br>1. The authors need to mention a previous publication describing the genome of P. tuberculanus: Lv J, Gao B, Liu P, Li J, Meng X. Linkage mapping aided by de novo genome and transcriptome assembly in Portunus trituberculatus: applications in growth-related QTL and gene identification. Sci Rep. 2017;7: 7874. doi:10.1038/s41598-017-08256-8). They need to compare their results with that of Lv et al., explain how they add to this previous knowledge, and discuss the discrepancies. The sentence at lines 70-71 should also be corrected, as it is not accurate that 'genomic research on the swimming crab has only been conducted at the transcriptome level, with the whole genome not yet described'.<br>Response: We appreciate this reviewer for the comments and suggestions. We have cited the paper (Lv et al., 2017) and corrected the sentence at lines 70-71 in the revised manuscript. Moreover, we also compared the published paper of Lv et al and the genome we assembled, and the results indicated that our genome assembly is obviously better than the published paper (Lv et al., 2017). This results were added in the revised supplementary files (Table S5). Thank you.<br><br>2. The manuscript identifies 50 chromosomes in P. tuberculanus genome, while linkage analysis of genomic markers lead Lv and colleagues to describe 53 linkage groups. The authors should discuss this discrepancy. In particular, it would be informative to map the 10000 markers used by Lv and colleagues to the proposed chromosomal assembly and compare to the genetic map.<br>Response: We appreciate this reviewer for the comments and suggestions. Using the 10,963 markers in Lv et al., 2017 paper, all these markers were mapped to our genome using blastn with e-value of 10-5. Then, we found that 10,897 markers (99.40%) can be found in our genome, which proved that our genome have high-quality and completeness. What's more, the 50 assembled chromosomes have 10,769 markers, which accounts for almost 98.83% of all the mapped markers. The other

scaffolds only have ~1.17% markers, and only three scaffolds (scaffold 205, scaffold 452, and scaffold 523) have more than 10 markers (including 10). Moreover, the length of these three scaffolds is quite short, which indicates that these three scaffolds may be part of some chromosomes, not a complete chromosome. So we think that the swimming crab genome should have 50 chromosomes. This table was added in the revised supplementary files (Table S6). Thank you.

3. Genome size estimation is performed on the sole basis of the position of maximal density of 17-mers in BGISeq-500 short reads. However, the k-mer curve presented in Figure S1 has an unusually flat shape, with a secondary peak at ~90 depth, which casts some doubt on the accuracy of this measure. Moreover, the proposed estimation (1 Gb) differs substantially from that made by Lv and colleagues, who put forward a size of 0.8 Gb based on the analysis of 23-mer frequency. Given the importance of this result for estimating the degree of completion of the proposed assembly, the authors need to provide a more solid genome size estimation, either by choosing an alternative method (eg flow cytometry), providing k-mer counts for higher k, or otherwise explaining both the unusual shape of their k-mer curve and the discrepancy between their estimation and the one in Lv et al., 2017.
Response: We appreciate this reviewer for the comments and suggestions. We also used k-mer frequency with k set as 23 to check the genome size this time, and the estimated genome size is 959,508,443 bp, which is very close with the assembled genome size. Due to the high rate of heterozygosity, Figure S1 has two peaks and the shape looks a little flat. But the accuracy of this measure is reliable, and the peak of heterozygosity (80) is exactly 1/2 of the main peak (160), indicating the estimated results is reliable.

4. The submitted manuscript lacks all figure legends. Although this is probably due to a mistake during the submission process, this makes the signification of some figures very difficult to understand. In particular Figure 4a is of no use as long as what is described as 'unclustered genes', 'unique paralogues', 'multiple copy orthologs' or 'other orthologs' is not defined. A revised submission must of course include descriptive figure legends.
Response: Sorry for this mistake, we have added the figure legends in the revised manuscript. Thank you.

5. It would be of interest to report the rate of heterozygosity found in the sequenced individual.
Response: The rate of heterozygosity in the sequenced swimming crab individual was calculated using k-mer and the rate is ~0.9%.

6. l 168-170 : the method for gene annotation using BLAST similarity with KEGG, SwissProt and TrEMBL databases should be more detailed : which BLAST program ? which parameter values ? which subject species ? which score threshold required to call an annotation ? Etc.
Response: Thank you for your suggestions. We have added the information you mentioned in the revised manuscript. Thank you.

7. l 99-100 : the sentence 'For the short reads, any reads with more than 10% unknown reads or 100 low-quality bases more than 50% along with its paired-end read were removed' is very obscure. Please clarify.
Response: Sorry for this misleading. We have corrected this in the revised manuscript.

8. l 216 : please define precisely the seldom-used term 'mounting rate'.
Response: The mounting rate means the total length of the contigs that anchored to chromosomes divided by the total length of all assembled contigs. We also add the description of 'mounting rate' in the revised manuscript.

9. l 268-273 : picking 3 'interesting' terms out of the list of 34 KEGG terms that show enrichment in the unique gene families is very little informative and even misleading, especially when these 3 terms appear rather far (ranks 20, 29 and 30) in this list. This paragraph, and possibly the enrichment analysis itself should be discarded : the genome assembly and annotation are interesting enough in their own respect without having to add such poorly supported speculations.
Response: Sorry for this misleading. We have removed this analysis (enrichment

analysis) in the revised manuscript. Thank you.

10. l 292-300 : The conclusions on the relative evolution rates are not appropriate. First, the authors should not use the ill-defined term 'survival pressure'. Second, it is oversimplistic to interpret faster or slower evolution rates in terms of selection, since these rates are influenced by many other factors, including mutation rates, population size, etc.
Response: Thank you for your suggestions. We have revised the words in this part (including 'survival pressure') in the revised manuscript.

11. The authors should remove any reference to 'adaptive evolution' from the manuscript title, since nothing in their data points to evidence of adaptive evolution.

Response: We have removed the words of 'adaptive evolution' in manuscript title in the revised manuscript. Thank you.

12. l 294 and 191-194: Please explain the choice of LINTRE, a rather uncommon tool in the field, to assess evolution rates. Please also provide more precise reference (the cited 1995 paper does not directly refer to a program), program version, choice of parameters.
Response: Thank you for your suggestions. LINTRE is a pretty old software but it is very useful, which also used in some other papers, such as in the NATURE paper "The seahorse genome and the evolution of its specialized morphology" (Lin et al.) that published in 2016. We used the version 1 of LINTRE and all the parameters were used as default. We also make a change in our manuscript. Thank you.

13. L302-311: the last paragraph of the results is highly speculative, with no interpretation as to why the crab-enriched signalling pathways (HIF1, Hippo and insulin) might be particularly relevant to the evolution of this species. Although factually correct, this analysis could easily be removed from the manuscript to lend more weight to its important parts: the assembly and annotation of the genome.
Response: Thank you for your suggestions. We have removed this part in the revised manuscript.

14. L492: Reference 48 has no author names.
Response: Sorry for this mistake, we have corrected it in the revised manuscript.

15. Availability of the data: in the case of a revised submission, it would be more convenient for reviewers if they could have access to the genome data in the same form that will be eventually available from GigaDB. At present, nothing can be accessed there.
Response: We have uploaded all the related data, including the data of genome assembly and annotation to GigaDB. Thank you.

Reviewer #2 Comments for the Author…
1. This paper describes a high-quality genome assembly of Portunus trituberculatus, one of the most widely fished species of crab in the world. The paper is written clearly and succinctly. The figures look excellent are clear and informative. I downloaded the genome and found an expected Hox cluster, which is in line with this being a high quality data set. The authors do not describe the Hox cluster and this is fine, but they should consider since it would not have to be that extensive of an analysis and a description plus Hox complex figure would increase the interest in the paper (it could also be a follow-up study). Nevertheless, this is a wonderful genomic resource, an excellent analysis, and if the authors address the reproducibility issues in my next paragraph, I would say that this is a model genome data note.
Response: Thank you for your suggestions. Yes, hox cluster annotation and hox gene evolution analysis are the next points in our follow-up study, and several crab species may be included. Thank you.

2. The methods appear thorough, however repeating these analyses in full would be impossible without guessing at some parameter settings etc. In order to make the work repeatable, please include ALL command lines in a supplemental document. There is an excellent example in the supplement linked here:
https://academic.oup.com/mbe/article/35/2/486/4644721#113627427

	Response: Thank you for your suggestions. In order to make all the parameter setting clear, we have added the parameter of the software in the revised manuscript refer to the applied example. Thank you.<br><br>3. Line 34: "only limited transcriptome data currently available"<br>--This is untrue as there is a draft genome assembly available in GenBank: https://www.ncbi.nlm.nih.gov/nuccore/VSRR000000000.1<br>This available draft should be acknowledged in the manuscript (even though the assembly in this current study is far superior).<br>Response: Sorry for this mistake. We have corrected this description and added the comparison between our genome and the published genome. This table was added in the revised supplementary files (Table S5). Thank you.<br><br>4. Line 70: "genomic research on the swimming crab has only been conducted at the transcriptome level [14-16], with the whole genome not yet described. --Likewise, this line should be updated to mention this draft genome.<br>Response: Sorry for this mistake. We have corrected this mistake in the revised manuscript.<br><br>5. Line 84: "Muscle RNA was also extracted using TRIzol (Invitrogen) according to the manufacturer's instructions" -- Should clarify whether the same animal used was used for extraction of RNA asthe genome. Indeed it should also be noted if the same animal was used for all genomic sequencing.<br>Response: Thank you for your suggestions. The same animal was used in all the RNA and DNA sequencing. We have corrected this in the revised manuscript.<br><br>6. Line 297 (and line 300): "greater survival pressures on these two species"<br>--I wouldn't attribute faster evolutionary rates to "survival pressures." Evolutionary rate has more to do with generation time (shorter=greater) and population size (larger=greater). The evolutionary rate makes sense in relation to both of these factors. Differences in survival pressures are heavily influenced by competition in large populations.<br>Response: Thank you for your suggestions. We have modified the inappropriate words in the revised manuscript.<br><br>7. Table 2: It seems as if the "Summary" row represents the percentage for "Complete BUSCO (C)." The label "Summary" does not make sense in this context. I would rename to "Summary (percentage Complete Busco)" or "percentage Complete Busco"<br>Response: Thank you for your suggestions. We have corrected this mistake in the revised manuscript.<br><br>8. I love Figure 1! Beautiful creature!<br>Response: Thank you.<br><br>9. Figure 2 and 3 legends should include more information. For example, what program was used to generate figure. What is the underlying data from, etc.<br>Response: Thank you for your suggestions. We have added more information to describe these two figures in the revised manuscript. Thank you.
**Additional Information:**	
**Question**	**Response**
Are you submitting this manuscript to a special series or article collection?	No
**Experimental design and statistics**<br><br>Full details of the experimental design and statistical methods used should be given in the Methods section, as detailed in our Minimum Standards Reporting Checklist. Information essential to interpreting the	Yes

data presented should be made available in the figure legends.<br><br>Have you included all the information requested in your manuscript?	
**Resources**<br><br>A description of all resources used, including antibodies, cell lines, animals and software tools, with enough information to allow them to be uniquely identified, should be included in the Methods section. Authors are strongly encouraged to cite Research Resource Identifiers (RRIDs) for antibodies, model organisms and tools, where possible.<br><br>Have you included the information requested as detailed in our Minimum Standards Reporting Checklist?	Yes
**Availability of data and materials**<br><br>All datasets and code on which the conclusions of the paper rely must be either included in your submission or deposited in publicly available repositories (where available and ethically appropriate), referencing such data using a unique identifier in the references and in the "Availability of Data and Materials" section of your manuscript.<br><br>Have you have met the above requirement as detailed in our Minimum Standards Reporting Checklist?	Yes

Manuscript

Click here to access/download;Manuscript;309-HL-author1.docx

Click here to view linked References

Chromosome-level genome assembly reveals the unique genome evolution of the swimming crab (*Portunus trituberculatus*)

Boping Tang[1,*], Daizhen Zhang[1,*], Haorong Li[2,*], Senhao Jiang[1], Huabin Zhang[1], Fujun Xuan[1], Baoming Ge[1], Zhengfei Wang[1], Yu Liu[1], Zhongli Sha[3], Yongxu Cheng[5], Wei Jiang[3], Hui Jiang[4,6], Zhongkai Wang[2], Kun Wang[2], Chaofeng Li[1], Yue Sun[1], Shusheng She[7], Qiang Qiu[2], Wen Wang[2], Xinzheng Li[3], Yongxin Li[2‡], Qiuning Liu[1†], Yandong Ren[2†]

1. Jiangsu Key Laboratory for Bioresources of Saline Soils, Jiangsu Provincial Key Laboratory of Coastal Wetland Bioresources and Environmental Protection, Jiangsu Synthetic Innovation Center for Coastal Bio-agriculture, Yancheng Teachers University, Yancheng 224002, China

2. Center for Ecological and Environmental Sciences, Northwestern Polytechnical University, Xi'an 710072, China

3. Institute of Oceanology, Chinese Academy of Sciences, Qingdao 266071, China

4. National Engineering Laboratory of Marine Germplasm Resources Exploration and Utilization, Zhejiang Ocean University, Zhoushan 316022, China

5. Key Laboratory of Freshwater Aquatic Genetic Resources, Ministry of Agriculture and Rural Affairs, Shanghai Ocean University, Shanghai 201306, China

6. National Engineering Research Center for Facilitated Marine Aquaculture, Zhejiang Ocean University, Zhoushan 316022, China

7. China Hong Kong Ecology Consultant Company, Hong Kong, China

*These authors contributed equally to this work.

†Corresponding authors: Yandong Ren (renyandong90@126.com); Qiuning Liu (liuqiuning@163.com).

‡Senior author: Yongxin Li (yxli28science@sina.com)

ORCIDs:

Yandong Ren: 0000-0003-1595-8994

Yongxin Li: 0000-0002-9555-1387
Wen Wang: 0000-0002-7801-2066
Zhongkai Wang: 0000-0003-0578-5735
Kun Wang: 0000-0001-6059-6529
Qiang Qiu: 0000-0002-9874-271X

Abstract

Background: The swimming crab, *Portunus trituberculatus*, is an important commercial species in China and is widely distributed in the coastal waters of Asia-Pacific countries. Despite increasing interest in swimming crab research, a high quality chromosome-level genome is still missing. **Findings:** Here, we assembled the first chromosome-level reference genome of *P. trituberculatus* by combining the short reads, Nanopore long reads, and Hi-C data. The genome assembly size was 1.00 Gb with a contig N50 length of 4.12 Mb. In addition, BUSCO assessment indicated that 94.7% of core eukaryotic genes were present in the genome assembly. Approximately 54.52% of the genome was identified as repetitive sequences, with a total of 16,796 annotated protein-coding genes. In addition, we anchored contigs into chromosomes and identified 50 chromosomes with a N50 length of 21.80 Mb by Hi-C technology. **Conclusions:** We anticipate that this chromosome-level assembly of the *P. trituberculatus* genome will not only promote study of basic development and evolution but also provide important resources for swimming crab reproduction.

Keywords: *Portunus trituberculatus*; genome assembly; crab; chromosome; evolution

Introduction

The swimming crab, *Portunus trituberculatus* (NCBI: txid210409, marinespecies.org:taxname:1061762), belonging to Brachyura, Portunidae, Portunus, is named for its shuttle-shaped head breastplate and three verrucous bumps on the back of the stomach and heart regions [1, 2]. The chelipeds of swimming crabs are well developed for feeding and attacking, with the first three pairs and last pair used for crawling and swimming, respectively [3, 4]. Male and female crabs are distinguished by their type of abdomen, with the male having a triangular abdomen and the female having an almost circular one [5]. Due to their lack of drilling ability, swimming crabs often live in soft mud or sand [6] or in

seagrass near the shore, and also show a certain level of phototaxis, spending time on the sea floor during the day and foraging at night [5]. Swimming crabs are also omnivorous, feeding on shellfish, small fish, shrimp, algae, and decomposing animal and plant carcasses [7].

The swimming crab is widely distributed in the coastal waters of Korea, Japan, China, and Southeast Asia and is one of the most valuable marine crustaceans in Asia [8] . It is widely found in Chinese coastal waters of the Bohai Sea, Yellow Sea, East China Sea, and South China Sea and is an important commercially cultured species [9]. Swimming crabs are considered highly nutritious, especially in regard to crab cream, and are very popular in China [10, 11]. As a result, the crab has been heavily overfished, resulting in substantial declines in its natural population [12] and initiation of artificial breeding [13, 14]. With continued research on the crab, it has become clear that morphological, physiological, but the genetic changes are poorly understood. At present, several studies of swimming crab on genomic research have been carried out [15-18], but the high-quality chromosome-level genome is still missing.

In the present study, we constructed a chromosome-level genome assembly of *P. trituberculatus* by combining short reads, Nanopore long reads, and Hi-C sequencing data. This chromosome-level genome will not only promote study on development and evolution, but also provide important resources for reproductive studies of *P. trituberculatus* and other crab species.

Sampling, library construction, and sequencing

A male swimming crab was collected in Bohai Bay, Hebei Province, China, for sequencing (Figure 1). To obtain sufficient high-quality DNA for the Oxford Nanopore (Oxford, UK) and BGISEQ-500 platforms (BGI, China), the swimming crab was rinsed five times with clean water and dissected immediately. Fresh muscle tissue was collected and snap-frozen in liquid nitrogen. The samples were then used to extract DNA with a Qiagen Blood & Cell Culture DNA Mini Kit and prepared for Nanopore, BGISEQ-500, and Hi-C sequencing. Using the same individual, muscle RNA was also extracted using TRIzol (Invitrogen) according to the manufacturer's instructions. To obtain an overview of the transcriptome, polyadenylated RNA was chosen by oligo (dT) purification and reverse-transcribed to cDNA and sequenced using the BGISEQ-500 platform.

Extracted DNA was sequenced using both the BGISEQ and Oxford Nanopore platforms. The

short reads generated from the BGISEQ platform were used for estimation of genome size and error correction of the assembled genome, and the Nanopore long reads were used for genome assembly. To this end, one library with insertion lengths of ~300 bp was sequenced on the BGISEQ-500 platform, and another library with an average length of 20 kb was constructed using the Oxford Nanopore platform according to the manufacturers' protocols.

Data filtering

Three different sources of reads were used to achieve the high-quality genome assembly, i.e., Nanopore long reads, short reads, and Hi-C reads. Thus, we used different methods for filtering. For the Nanopore long reads, any reads less than 1 kb or with a mean quality value of < 7 were removed. For the short reads, any read with more than 10% unknown bases (usually stand by "N") or with more than 50% low-quality bases were removed, and its paired-end read was also removed. All adaptor sequences and duplicated reads produced by polymerase chain reaction (PCR) were removed. The low-quality Hi-C reads were filtered using HiC-Pro v2.10.0 [19] with default parameters..

Genome characteristic estimation

All filtered BGISEQ short reads were used for estimation of genome size and other characteristics. In addition, 17-mer was chosen for k-mer analysis and the 17-mer depth frequency distribution was calculated using the k-mer method. Genome size was estimated as: Genome size = TKN17-mer / PKFD17-mer, where TKN17-mer is the total k-mer number and PKFD17-mer is the peak k-mer frequency depth of 17-mer. The estimated genome size was used to determine subsequent genome assembly results.

Genome assembly

To improve the quality of the genome and reduce the error ratio, self-error correction of all Nanopore long reads was performed using NextDenovo software [20]. The error-corrected Nanopore long reads were then used to assemble the raw genome via contig construction with WTDBG software (WTDBG, RRID:SCR_017225) [21] and parameters: -p 0 -k 15 -AS 2 -E 1

-s 0.05 -L 5000. The assembled genomic sequences were further polished by Racon v1.2.1 [22] with four iterations using all the error-corrected Nanopore long reads with default parameters. After this, all filtered BGISEQ short reads were polished by Pilon v1.21 (Pilon, RRID:SCR_014731) [23] at the single-base level with default parameters. After completion of the error-correction steps, the Hi-C data were used to obtain a chromosome-level genome assembly. All Hi-C sequencing data were first filtered by Hic-Pro v2.10.0 [19] with default parameters and then mapped to the polished swimming crab genome to improve the connection integrity of the contigs. Finally, 3D *de novo* assembly software (v180419) [24] with default parameters was used to determine contig location and direction.

Genome assembly evaluation

Three different strategies were used to evaluate the completeness and accuracy of the assembled genome. First, the quality of the assembled genome and gene completeness were assessed using BUSCO (BUSCO, RRID:SCR_015008) [25] with the core gene sets of the eukaryote and metazoan databases, respectively. Second, all filtered short reads generated by BGISEQ were mapped to the assembled genome using BWA-MEM v0.7.12 [26] to detect genome integrity with default parameters. Third, transcripts were mapped to the assembled genome using BLAT software (BLAT, RRID:SCR_011919) [27] with e value less than 10-5.

Repetitive element annotation

Tandem repeats and transposable elements (TEs) were also annotated in the chromosome-level genome. Tandem repeats were annotated using Tandem Repeat Finder v4.04 [28] with default parameters. The TEs were annotated at the protein level using RepeatProteinMask (RM-BLASTX) to search the protein database and at the DNA level using RepeatMasker (open-4.0.7, RepeatMasker, RRID:SCR_012954) [29] to search the *de novo* libraries and repbase. The *de novo*-repeat libraries were constructed using RepeatModeler (RepeatModeler, RRID:SCR_015027) [30], with consensus sequences used for *de novo* library construction and all software were using the default parameters.

Gene structure prediction and function annotation

After repetitive element annotation, the repeat-masked genome was used for gene set annotation with three different methods, i.e., *de novo* prediction, RNA-seq-based annotation, and homology-based annotation. We first assembled the RNA-seq reads into transcripts using Bridger r2014-12-01 (Bridger, RRID:SCR_017039) [31]. The assembled genome and transcripts were then used for Augustus training to obtain an accurate Augustus annotation species model. Augustus v2.5.5 (Augustus, RRID:SCR_008417) [32] was used for *de novo* prediction of coding genes with the previous training results. Second, proteins of *Bicyclus anynana* (GCF_900239965.1) [33], *Bombus terrestris* (GCF_000214255.1) [34], *Drosophila melanogaster* (GCA_000001215.4) [35], *Mus musculus* (GCF_000001635.26) [36], *Stegodyphus mimosarum* (GCA_000611955.2), *Penaeus vannamei* (GCA_003789085.1), *Mesobuthus martensii* [37], *Eriocheir japonica sinensis* (i.e., *Eriocheir sinensis*) (GigaDB: 100186) [38-43], and *Tachypleus tridentatus* (GCA_004102145.1) [44] were downloaded from the NCBI, GigaDB, or their own databases. The longest transcript of each gene was selected for further annotation and phylogenetic analysis. All filtered genes were searched with an e-value cutoff of 1e-5, with the *blast* results then formatted and prepared for Genewise [45] prediction of the gene structure of the swimming crab genome. Third, for the RNA-seq-based method, all assembled transcripts were aligned against the genome using BLAT [27] (identity >90% and coverage >90%), with PASA used to filter overlaps to link the spliced alignments. Finally, EvidenceModeler (EVM; EVidenceModeler, RRID:SCR_014659) v1.1.1 was used to integrate the above data into an EVM-derived gene set [46].

Five different public protein databases were used for gene functional annotation of the swimming crab, with InterProScan v4.8 (InterProScan, RRID:SCR_005829) [47] used to screen proteins against the five databases (Pfam, release 27.0, PRINTS, release 42.0, PROSITE, release 20.97, ProDom, 2006.1, and SMART, release 6.2) to determine the number of InterPro and GO predicted protein-coding genes. In addition, the Kyoto Encyclopedia of Genes and Genomes, UniProt/SwissProt, and UniProt/TrEMBL databases were also used for functional annotation with BLAST v2.3.0 [48]. Blastp (BLASTP, RRID:SCR_001010) was used in this step, and the e value was set as 10^{-5} and other parameters were set as defaults.

Identification of orthologous genes

The annotated genes in the swimming crab and six other species, including *Aedes aegypti* (GCF_002204515.2), *B.anynana*, *D. melanogaster*, *S. mimosarum*, *P.vannamei*, and *E. j. sinensis*, were used for orthologous gene identification with OrthoMCL v2.0.9 [49] with default parameters. The identified genes were then used to run reciprocal alignment and pairwise relationship analysis. The reciprocal best similarity pairs in different species were considered as putative orthologous genes and reciprocal better similarity pairs in one species were considered as paralogous genes. The 1:1:1:1:1:1:1 single-copy genes in the seven species were also identified for further phylogenetic and divergence time estimation analysis.

Phylogenetic analysis and divergence time estimation

Using the single-copy genes of the seven species (*P. trituberculatus*, *A. aegypti*, *B. anynana*, *D. melanogaster*, *S. mimosarum*, *P. vannamei*, and *E. j. sinensis*), we connected the genes in each species into one super-gene for phylogenetic tree building. Maximum likelihood-based phylogenetic analysis was conducted using RAxML v8.2.10 (RAxML, RRID:SCR_006086) [50] with default parameters. The MCMCTREE program in the PAML package v4.8 [51] was then used to calculate divergence time, with all fossil records downloaded from the TIMETREE website [52] for calibration.

Relative evolution rate

The relative evolution rate of species was analyzed with LINTRE software (version 1) [53] using the *tpcv* model and *S .mimosarum* as an outgroup. Using the default parameters of LINTRE, we then evaluated the relative evolution rate between the swimming crab and other related species.

Gene family expansion and contraction

Using the divergence time results calculated by MCMCTREE and the gene pairwise relationships calculated by OrthoMCL [49], we determined gene family expansion and

contraction for each node using CAFÉ v3.1 (CAFÉ, RRID:SCR_005983) [54]. The expansion and contraction genes of the swimming crab were extracted for GO/KEGG enrichment analysis [55, 56].

Results

Chromosome level genome assembly

To obtain a high-quality chromosome-level swimming crab genome, we extracted high-quality DNA from the muscle tissue and constructed libraries for genome sequencing. To estimate the genome characteristics of the swimming crab, we generated 205.40 Gb of BGISEQ data (Additional File: Table S1), with 17-mer analysis indicating a genome size of ~918.52Mb and the heterozygosity rate is ~0.9% (Additional File: Figure S1). In total, we generated 54.97 Gb (54.75-fold coverage) of Nanopore long read data with N50 over 20kb (Additional File: Table S2). The Nanopore long reads were assembled into contigs using WTDBG software [21] (genome size: 1.00 Gb; N50: 4.12 Mb) (Table 1). To further improve genome accuracy, we aligned all corrected Nanopore long reads to the assembled genome and conducted error-correction using Racon [22] with four iterations. The genome was subsequently corrected using all filtered BGISEQ clean reads via Pilon [23] with two iterations. We then constructed the chromosome-level genome with 95.95 Gb of Hi-C sequencing data (Additional File: Table S3) by 3D *de novo* assembly [24]. Finally, we obtained 50 chromosomes and a mounting rate (total length of the contigs that anchored to chromosomes divided by the total length of all assembled contigs) of 97.80% (Figure 2; Additional File: Table S4), which is the first chromosome-level crab genome with N50 of 21.79 Mb (Table 1). The high mounting rate suggested successful assembly of the swimming crab genome at the chromosome level. We also compared our assembled genome to the published swimming crab genome, the assembly quality of our genome is better than the previous one (Table S5). Due to the previous study has the genomic markers, we also mapped all the markers to our genome, and found that 99.40% (10,897 of 10,963) markers can be mapped to our genome. Among these mapped genome marker, 98.83% (10,769 of 10,897) are exactly mapped to our assembled 50 chromosomes (Table S6). All these results shown that, we obtained a high quality and quite complete chromosome-level genome.

Genome quality evaluation

We next assessed the completeness of the swimming crab genome by BUSCO [25] and identified 94.7% Eukaryota and 92.9% Metazoa conserved core genes in the genome (Table 2). We checked the mapping rates of the BGISEQ short reads to our genome and found that 95.85% of reads were properly pair-mapped to the genome (Additional File: Table S7). We then *de novo* assembled the transcripts using the RNA-seq data (Additional File: Table S8) with Bridger software [31] and a N50 length of 2,124 bp (Additional File: Table S9). After transcript mapping, we found that 97.80% of the transcripts could be mapped to the swimming crab genome (Additional File: Table S10). We also analyzed the genome quality of previously published high-quality genomes from closely related species and determined that the quality of the assembled chromosome-level swimming crab genome was markedly higher or comparable with that of other species (Additional File: Table S11). In summary, these results indicated that we acquired a high-quality swimming crab genome. To investigate genome characteristics, such as GC content, we analyzed the GC distribution in the genome with a slide-window method. The peak value of GC content was ~41%, which agrees with the average GC content in the swimming crab genome. We also found that the GC content in the swimming crab was closer to that of mouse than of shrimp (Additional File: Figure S2).

Genome annotation

The repetitive sequences of the swimming crab genome were identified through four different methods, resulting in 547.39 Mb of repeated sequences and accounting for 54.52% of the assembled genome (Additional File: Table S12). Among the repeated sequences, 19.28% (~193.56 Mb) were tandem repeats and 52.29% (~525.49 Mb) were TEs (Additional File: Table S12; Table 3). The TEs could be further divided into four main types, including 0.014% (~142.88kb) of short interspersed elements (SINE), 15.23% (~153.03 Mb) of long interspersed elements (LINE), 14.90% (~149.71 Mb) of DNA elements, and 4.50% (~45.19 Mb) of long terminal repeats (LTR) (Table 3).

After masking the repeated sequences, we annotated the protein-coding genes using *de novo*

prediction, homology-based prediction, and transcript-based prediction. We merged the results and obtained 16,791 protein-coding genes. We checked the quality of the annotated genes by comparing with several closely related species. Results showed that the mRNA, CDS, exon, intron length distributions of the swimming crab were similar to those of the closely related species, suggesting that the swimming crab annotation results were dependable (Figure 3).

We also performed functional annotation of the 16,791 genes with InterPro, GO, KEGG, SwissProt, and TrEMBL. The highest annotation rate (74.77%) was found for SwissProt, in which 12,558 genes were annotated. In total, 16,053 genes (~95.58%) were annotated, indicating that most genes could be found in the public protein databases (Table 4). Thus, taken together, we acquired a high-quality protein-coding gene set for the swimming crab.

Orthologous identification and gene family analysis

For comparative genomics analysis of the swimming crab, we analyzed the orthologous gene relationships among several species, including *A. aegypti*, *B. anynana*, *D. melanogaster*, *S. mimosarum*, *P. vannamei*, and *E. j. sinensis* using OrthoMCL. In total, 15,503 gene families were clustered in the seven species and 1,018 one-to-one single-copy genes were identified (Figure 4A). Because the swimming crab has several unique characteristics, we employed gene family analysis and found 8,832 gene families shared among the seven species, with 328 gene families unique to the swimming crab (Figure 4B). We then employed functional analysis and identified 34 enriched KEGG terms (Additional File: Table S13), suggesting these unique gene families play important roles in the swimming crab.

Phylogenetic relationships and divergence time

Although the phylogenetic relationships of the swimming crab and closely related species have been analyzed in previous studies, most used few nuclear and mitochondrial genes. To determine the evolutionary relationship of the swimming crab, we analyzed all single-copy genes using RAxML software [50], with the spider used as the outgroup species. Results showed that the swimming crab has a close relationship with the Chinese mitten crab and shrimp (Figure 5A). The seven species of pancrustaceans—*P. trituberculatus*, *A. aegypti*, *B.*

anynana, D. melanogaster, S. mimosarum, P. vannamei, and *E. j. sinensis*—formed two clades: i.e., Hexapoda and Crustacea. The Hexapoda group consisted of all lepidopteran and dipterous insects, whereas the second clade comprised all other crustaceans, with *P. trituberculatus* and *E. j. sinensis* forming a Pleocyemata clade, followed by Dendrobranchiata shrimp (*P. vannamei*). In addition, Hexapoda and Crustacea were both found to be monophyletic (Figure 5A). To determine divergence time, we employed MCMCTREE analysis in the PAML package [51] and found that the Chinese mitten crab and swimming crab diverged ~183.5 million years ago (Mya), and diverged from shrimp ~428.5 Mya (Figure 5A).

Relative evolution rate

Species in different environments can experience different survival pressures. As such, we conducted relative evolution rate analysis in LINTRE (version 1) [53], with spider as the outgroup species and swimming crab as the reference species. Results showed that the shrimp had the slowest evolution rate among the seven species, whereas the fruit fly and butterfly exhibited relatively fast evolution rates (Figure 5B; Additional File: Table S14). Interestingly, the slowest evolution rates were found among the Malacostraca (Figure 5B; Additional File: Table S14), suggesting the specific environments or habitats caused the different evolution rates of them.

Gene family expansion and contraction

We performed gene family expansion and contraction analysis of the seven species using CAFÉ v4.0, and identified 148 and 25 expanded and contracted gene families ($P < 0.05$) in the swimming crab, respectively. We then employed KEGG functional enrichment analysis of the expanded gene families and found that the HIF-1 signaling pathway (*Q*-value = 0.000109025), focal adhesion (*Q*-value = 0.000135977), Hippo signaling pathway (*Q*-value = 0.000184649), and insulin signaling pathway (*Q*-value = 0.000357592) were enriched (Additional File: Table S15). These biological processes are related to early development, hypoxia adaptation, and other key processes, which may help us better understand the evolution of swimming crab.

Conclusions

Based on BGISEQ, Nanopore, and Hi-C sequencing data, we assembled a chromosome-level high-quality genome of the swimming crab. Evaluation results indicated that the genome quality of swimming crab was comparable with that of most high-quality model species. We also successfully obtained 16,791 high-quality protein-coding genes by integrating three different methods. The genome and annotation data will help researchers better understand the evolution of crabs and improve their economic value. The phylogenetic results indicated that the swimming crab is closely related to the Chinese mitten crab, from which it diverged ~183.5 Mya. The unique and/or expanded gene family analysis provides clues to swimming crab development and environmental adaptation.

Availability of supporting data

The raw sequencing data were deposited in the NCBI database under accession number PRJNA555262. The genome assembly and annotation results are available via the *GigaScience* repository GigaDB [57].

Additional files

Table S1: Statistics on genome sequencing data from BGISEQ platform.

Table S2: Statistics on sequencing reads from Oxford Nanopore platform.

Table S3: Statistics on Hi-C sequencing data.

Table S4: Statistics on assembled chromosome-level genome by 3D *de novo* assembly software.

Table S5. The quality comparison of these two genomes.

Table S6. The mapping results of genomic markers to the assembled genome.

Table S7: Statistics on mapping ratio of the BGISEQ short reads to swimming crab genome.

Table S8: Statistics on RNA-seq data.

Table S9: Statistics on assembled transcripts by Bridger software.

Table S10: Statistics on transcript mapping ratio of swimming crab genome.

Table S11: Genome quality comparison of swimming crab with other species.

Table S12: Statistics on annotated repetitive sequences using different software.

Table S13: KEGG enrichment analysis of unique gene families in swimming crab relative to

six other species.

Table S14: Two-cluster analysis of swimming crab and other species.

Table S15: KEGG enrichment analysis of expanded gene families in swimming crab.

Figure S1: 17-mer analysis of swimming crab genome.

Figure S2: GC distribution in species.

Abbreviations

Hi-C: High-throughput chromosome conformation capture; BUSCO: Benchmarking Universal Single-Copy Orthologs; CDS: Coding DNA Sequence; DNA: Deoxyribonucleic Acid; RNA: Ribonucleic Acid; RNA-seq: RNA sequencing; BLAST: Basic Local Alignment Search Tool; KEGG: Kyoto Encyclopedia of Genes and Genomes; NCBI: National Center for Biotechnology Information.

Conflicts of interest

The authors declare that they have no competing interests.

Funding

This study was supported by the National Natural Science Foundation of China (31672267, 31640074), Jiangsu Agriculture Science and Technology Innovation Fund (CX(18)3027, CX(18)2027), Natural Science Foundation of Jiangsu Province (BK20171276, BK20160444), “Qing Lan Project” of Daizhen Zhang and China Postdoctoral Science Foundation (2018M642105).

Author contributions

Y.R., Q.L., Yongxin L., and X.L. conceived the project. B.T., D.Z., S.S., H.Z., Yu L., and S.J. collected and dissected the samples. H.L., Zhongkai W., K.W., Y.S., Q.Q., C.L., and Yongxin L. estimated genome size. F.X., Y.C., W.J., and H.J. assembled the genome. B.G., Zhengfei W., Z.S., and B.T. performed genome assembly, genome annotation, and evolution analysis. Y.L., B.T., Q.Q., and W.W. wrote the manuscript. Y.R. and W.W. revised the manuscript.

REFERENCES

1. Spiridonov VA, Neretina TV and Schepetov D. Morphological characterization and molecular phylogeny of Portunoidea Rafinesque, 1815 (Crustacea Brachyura): Implications for understanding evolution of swimming capacity and revision of the family-level classification. Zoologischer Anzeiger - A Journal of Comparative Zoology. 2014;253 5:404-29.
2. Dai A, Yang S and Song Y. Marine crabs in China Sea. Beijing: Marine Publishing Company; 1986.
3. Okamoto K. Malformed regeneration of partly cut swimming leg as a marker for swimming crab Portunus trituberculatus. Fisheries Science. 2010;72 5:1121-3.
4. Hazlett, B.A. (1971). Interspecific Fighting in Three Species of Brachyuran Crabs from Hawaii. Crustaceana, 20(3), 308-314. www.jstor.org/stable/20101793
5. Xue J, Du N, Wei L and Wu H. A review of studies on portunus trituberculatus in China. Dongahi Marineence. 1997.
6. Sakai T. Crabs of Japan and the Adjacent Seas. Crabs of Japan & the Adjacent Seas. 1976.
7. Wu RSS and Shin PKS. Food segregation in three species of portunid crabs. Hydrobiologia. 1997;362 1-3:107-13.
8. Marine Species Identification Portal: *Portunus trituberculatus* http://species-identification.org/species.php?species_group=crabs_of_japan&menuentry=soorten&id=1106&tab=beschrijving.

9. Qi JB, Gu XL, Ma LB, Qiao ZG and Chen K. The research progress on food organism culture and technology utilization in crab seed production in ponds in China. Agricultural Sciences. 2013;4 10:563-9.

10. Su XR, Li TW, Ding MJ and Chien PK. Evalution on nutritive value of *Portunus trituberculatus*. Chinese Journal of Oceanology & Limnology. 1997;15 2:168-72.

11. Wang Z, Sun L, Guan W, Zhou C, Tang B, Cheng Y, et al. De novo transcriptome sequencing and analysis of male and female swimming crab (*Portunus trituberculatus*) reproductive systems during mating embrace (stage II). BMC Genetics. 2018;19 1:3.

12. Dan S, Oshiro M, Ashidate M and Hamasaki K. Starvation of Artemia in larval rearing water affects post-larval survival and morphology of the swimming crab, *Portunus trituberculatus* (Brachyura, Portunidae). Aquaculture. 2016:S0044848615300442.

13. Hamasaki K, Obata Y, Dan S and Kitada S. A review of seed production and stock enhancement for commercially important portunid crabs in Japan. Aquaculture International. 2011;19 2:217-35.

14. Liu S, Sun J and Hurtado LA. Genetic differentiation of Portunus trituberculatus, the world\"s largest crab fishery, among its three main fishing areas. Fisheries Research. 2013;148 148:38-46.

15. Lv J, Ping L, Yu W, Baoquan G, Ping C, Jian L, et al. Transcriptome Analysis of *Portunus trituberculatus* in Response to Salinity Stress Provides Insights into the Molecular Basis of Osmoregulation. Plos One. 2013;8 12:e82155.

16. Yang Y, Wang J, Han T, Liu T, Wang C, Xiao J, et al. Ovarian Transcriptome

Analysis of Portunus trituberculatus Provides Insights into Genes Expressed during Phase III and IV Development. Plos One. 2015;10 10:e0138862.

17. Meng X, Liu P, Jia F, Li J and Gao BQ. Correction: De novo Transcriptome Analysis of *Portunus trituberculatus* Ovary and Testis by RNA-Seq: Identification of Genes Involved in Gonadal Development. Plos One. 2015;10 7:e0133659.

18. Lv J, Gao B, Liu P, Li J and Meng X. Linkage mapping aided by de novo genome and transcriptome assembly in *Portunus trituberculatus*: applications in growth-related QTL and gene identification. Scientific Reports. 2017;7 1:7874.

19. Servant N, Varoquaux N, Lajoie B, Viara E, Chen CJ, Vert JP, et al. HiC-Pro: an optimized and flexible pipeline for Hi-C data processing. Genome Biology. 2015;16 1:259.

20. "NextDenovo.". Nextomics, GitHub Repository. https://github.com/Nextomics/NextDenovo/. Nextomics. 2019.

21. Ruan J and Li H. Fast and accurate long-read assembly with wtdbg2. 2019.

22. Talay AC and Altilar DT. RACON: a routing protocol for mobile cognitive radio networks. In: *Acm Workshop on Cognitive Radio Networks* 2009.

23. Walker BJ, Abeel T, Shea T, Priest M, Abouelliel A, Sakthikumar S, et al. Pilon: An Integrated Tool for Comprehensive Microbial Variant Detection and Genome Assembly Improvement. Plos One. 2014;9 11:e112963.

24. Dudchenko O, Batra SS, Omer AD, Nyquist SK, Hoeger M, Durand NC, et al. De novo assembly of the *Aedes aegypti* genome using Hi-C yields chromosome-length scaffolds. Science. 2017;356 6333:92-5.

25. Simao FA, Waterhouse RM, Ioannidis P, Kriventseva EV and Zdobnov EM. BUSCO: assessing genome assembly and annotation completeness with single-copy orthologs. Bioinformatics. 2015;31 19:3210-2.

26. Li H and Durbin R. Fast and accurate short read alignment with Burrows-Wheeler transform. Bioinformatics. 2009;25 14:1754-60. doi:10.1093/bioinformatics/btp324.

27. Kent WJ. BLAT - The BLAST-like alignment tool. Genome Research. 2002;12 4:656-64.

28. Benson and G. Tandem repeats finder: a program to analyze DNA sequences. Nucleic Acids Research. 1999;27 2:573-80.

29. Bedell JA, Korf I and Gish W. MaskerAid : a performance enhancement to RepeatMasker. Bioinformatics. 2000;16 11:1040.

30. RepeatModeler. http://www.repeatmasker.org/RepeatModeler.

31. Chang Z, Li G, Liu J, Zhang Y, Ashby C, Liu D, et al. Bridger: a new framework for de novo transcriptome assembly using RNA-seq data. Genome Biology. 2015;16 1:30.

32. Stanke M and Waack S. Gene prediction with a hidden Markov model and a new intron submodel. Bioinformatics. 2003;19 suppl_2:215--25.

33. Nowell RW, Elsworth B, Oostra V, Zwaan BJ and Blaxter M. A high-coverage draft genome of the mycalesine butterfly *Bicyclus anynana*. GigaScience. 2017;6 7:1-7.

34. Sadd BM, Barribeau SM, Bloch G, de Graaf DC, Dearden P, Elsik CG, et al. The genomes of two key bumblebee species with primitive eusocial organization. Genome Biology. 2015;16 1:76. doi:10.1186/s13059-015-0623-3.

35. Adams MD, Celniker SE, Holt RA, Evans CA, Gocayne JD, Amanatides PG, et al.

The Genome Sequence of Drosophila melanogaster. Science. 2000;287 5461:2185.

36. Waterston RH, Lindblad-Toh K, Birney E, Rogers J, Abril JF, Agarwal P, et al. Initial sequencing and comparative analysis of the mouse genome. Nature. 2002;420 6915:520-62. doi:10.1038/nature01262.

37. Ma J and Shi YB. The *Mesobuthus martensii* genome reveals the molecular diversity of scorpion toxins. Cell & bioscience. 2014;4 1:1. doi:10.1186/2045-3701-4-1.

38. Linsheng S, Chao B, Yongju L, Lingling W, Xinxin Y, Jia L, et al. Draft genome of the Chinese mitten crab,*Eriocheir sinensis.* GigaScience. 2016;5 1:5.

39. Liu RY. Introduction to the classification of recent crustacean. In: Transactions of the Chinese crustacean society No. 4. The Chinese crustacean Society, Science Press, Beijing. 2003;No. 4:76-86.

40. Tang B, Zhou K, Song D, Yang G and Dai A. Molecular systematics of the Asian mitten crabs, genus Eriocheir (Crustacea: Brachyura). Molecular Phylogenetics & Evolution. 2003;29 2:309-16.

41. Song D. X. SLY. The Crustacea fauna of Hebei. Hebei Science and Technology Publishing House, Shijiazhuang. 2009:1-772.

42. Tang BP, Xin ZZ, Liu Y, Zhang DZ, Wang ZF, Zhang HB, et al. The complete mitochondrial genome of *Sesarmops sinensis* reveals gene rearrangements and phylogenetic relationships in Brachyura. 2017;12 6:e0179800. doi:10.1371/journal.pone.0179800.

43. Xuan F WX, Liu N, et al. Reproductive potential of individual male Chinese mitten crabs *Eriocheir japonica sinensis* in a local pond-reared broodstock: Implications for

parent crab selection and sex ratio optimization. Aquac Res. 2018;49:3498–507.

44. Gong L, Fan G, Ren Y, Chen Y, Qiu Q, Liu L, et al. Chromosomal level reference genome of Tachypleus tridentatus provides insights into evolution and adaptation of horseshoe crabs. 2019;19 3:744-56. doi:10.1111/1755-0998.12988.

45. Birney E and Durbin R. Using GeneWise in the Drosophila Annotation Experiment. Genome Research. 2000;10 4:547-8.

46. Haas BJ, Salzberg SL, Zhu W and Pertea M. Automated eukaryotic gene structure annotation using EVidenceModeler and the Program to Assemble Spliced Alignments. Genome Biology. 2008;9 1:R7.

47. Zdobnov EM and Apweiler R. InterProScan - an integration platform for the signature-recognition methods in InterPro. Bioinformatics. 2001;17 9:847-8.

48. Altschul SF. Basic local alignment search tool (BLAST). Journal of Molecular Biology. 2012;215 3:403-10.

49. Li and L. OrthoMCL: Identification of Ortholog Groups for Eukaryotic Genomes. Genome Research. 2003;13 9:2178-89.

50. Stamatakis and A. RAxML version 8: a tool for phylogenetic analysis and post-analysis of large phylogenies. Bioinformatics. 2014;30 9:1312-3.

51. Yang and Ziheng. PAML: a program package for phylogenetic analysis by maximum likelihood. Computer Applications in the Biosciences Cabios. 1997;13 5:555.

52. TIMETREE. http://www.timetree.org.

53. Takezaki N, ., Rzhetsky A, . and Nei M, . Phylogenetic test of the molecular clock and linearized trees. Molecular Biology & Evolution. 1995;12 5:823-33.

54. De Bie T, Cristianini N, Demuth JP and Hahn MW. CAFE: a computational tool for the study of gene family evolution. Bioinformatics. 2006;22 10:1269-71.

55. Beissbarth T and Speed TP. GOstat: find statistically overrepresented Gene Ontologies within a group of genes. Bioinformatics. 2004;20 9:1464-5.

56. Huang DW, Sherman BT and Lempicki RA. Bioinformatics enrichment tools: paths toward the comprehensive functional analysis of large gene lists. Nucleic Acids Research. 2009;37 1:1.

57. Ren Y; Tang B; Li H; Jiang S; Xuan F; Ge B; Wang Z; Liu Y; Sha Z; Cheng Y; Jiang W; Jiang H; Wang Z; Wang K; Li C; Sun Y; She S; Qiu Q; Wang W; Li X; Li Y; Liu Q (2019): Supporting data for "Chromosome-level genome assembly reveals adaptive evolution of the swimming crab (Portunus trituberculatus)" *GigaScience Database*. http://dx.doi.org/10.5524/100678

Table 1: Assembly of swimming crab genome.

Term	Contig phase		Hi-C phase	
	Size (bp)	Number	Size (bp)	Number
N90	439,683	334	11,273,125	41
N80	1,225,551	203	14,151,211	33
N70	2,035,154	141	16,942,622	27
N60	2,950,146	100	19,786,189	21
N50	4,121,416	71	21,793,880	17
Max length	17,984,318	-	42,710,960	-
Total length	1,004,084,521	-	1,005,046,021	-
Number>=100bp	-	2446	-	523
Number>=10kb	-	1756	-	314

Note: Contig phase represents results assembled by WTDBG software, and Hi-C phase represents scaffold statistics of genome after chromosome assembly.

Table 2: Quality evaluation of assembled swimming crab genome by BUSCO.

Library	Eukaryota	Metazoa
Complete BUSCO (C)	287	909
Complete and single-copy BUSCO (S)	283	903
Complete and duplicated BUSCO (D)	4	6
Fragmented BUSCO (F)	2	19

Missing BUSCO (M)	14	50
Total BUSCO groups searched	303	978
Percentage of complete BUSCO	94.7%	92.9%

Table 3: Statistics on transposable elements (TEs) in swimming crab genome.

Type	RepbaseTEs		TE proteins		*De novo*		Combined TEs	
	Length (bp)	% in genome	Length (bp)	% in genome	Length (bp)	% in genome	Length (bp)	% in genome
DNA	131,799,733	13.11%	2,434,533	0.24%	19,288,080	1.92%	149,711,951	14.90%
LINE	16,171,649	1.61%	75,759,827	7.54%	131,530,457	13.09%	153,027,744	15.23%
SINE	142,878	0.01%	0	0	0	0	142,878	0.014%
LTR	26,546,055	2.64%	10,195,324	1.01%	18,421,957	1.83%	45,189,365	4.50%
Other	89,969,319	8.95%	0	0	211,157,523	21.01%	230,116,216	22.90%
Unknown	34,752	0.0035%	0	0	90,989,908	9.05%	91,007,921	9.06%
Total	213,558,503	21.25%	88,375,336	8.79%	464,908,824	46.26%	525,492,271	52.29%

Table 4: Functional annotation of predicted protein-coding genes.

Term	Gene number	Percentage (%)
GO	8,712	51.87
InterPro	11,691	69.61
KEGG	10,880	64.78
SwissProt	12,558	74.77
TrEMBL	12,256	72.97
Annotated	16,053	95.58
Unannotated	743	4.42
Total	16,796	100

Figure legends

Figure 1: Swimming crab, *Portunus trituberculatus*. The adult male swimming crab collected from Bohai Bay, Hebei Province.

Figure 2: Genome characteristics of swimming crab. From outer circle to inner circle: gene distribution, tandem repeats (TRP), long tandem repeats (LTR), long interspersed nuclear elements (LINE) and the short interspersed nuclear elements (SINE), the DNA elements, and the GC content of the genome.

Figure 3: Annotation quality comparison of protein-coding genes. We compared the

mRNA length, CDS length, exon length and intron length among these species, including: P. trituberculatus, *A. aegypti*, *S. mimosarum*, *D. melanogaster*, and *P. vannamei*.

Figure 4: Gene family analysis of swimming crab. A. Orthologous genes among species. The multiple copy orthologs are orthologs have multiple copy in one species, the single copy orthologs are orthologs have only one copy in one species, the other orthologs are the rest orthologs, the unclustered genes are genes have no homology with others, the unique paralogs are genes only exists in one specific species. B: Unique and common gene families among these species, including: *B. anynana*, *A. aegypti*, *D. melanogaster*, *P. vannamei*, *E. j. sinensis*, *S. mimosarum*, and *P. trituberculatus*.

Figure 5: Phylogenetic relationships, divergence time, and evolution rate analysis. A. Phylogenetic relationship and divergence time of species. Red dot represents fossil record used here. B. Relative evolution rate of species.

Click here to access/download;Figure;Figure 1.pdf

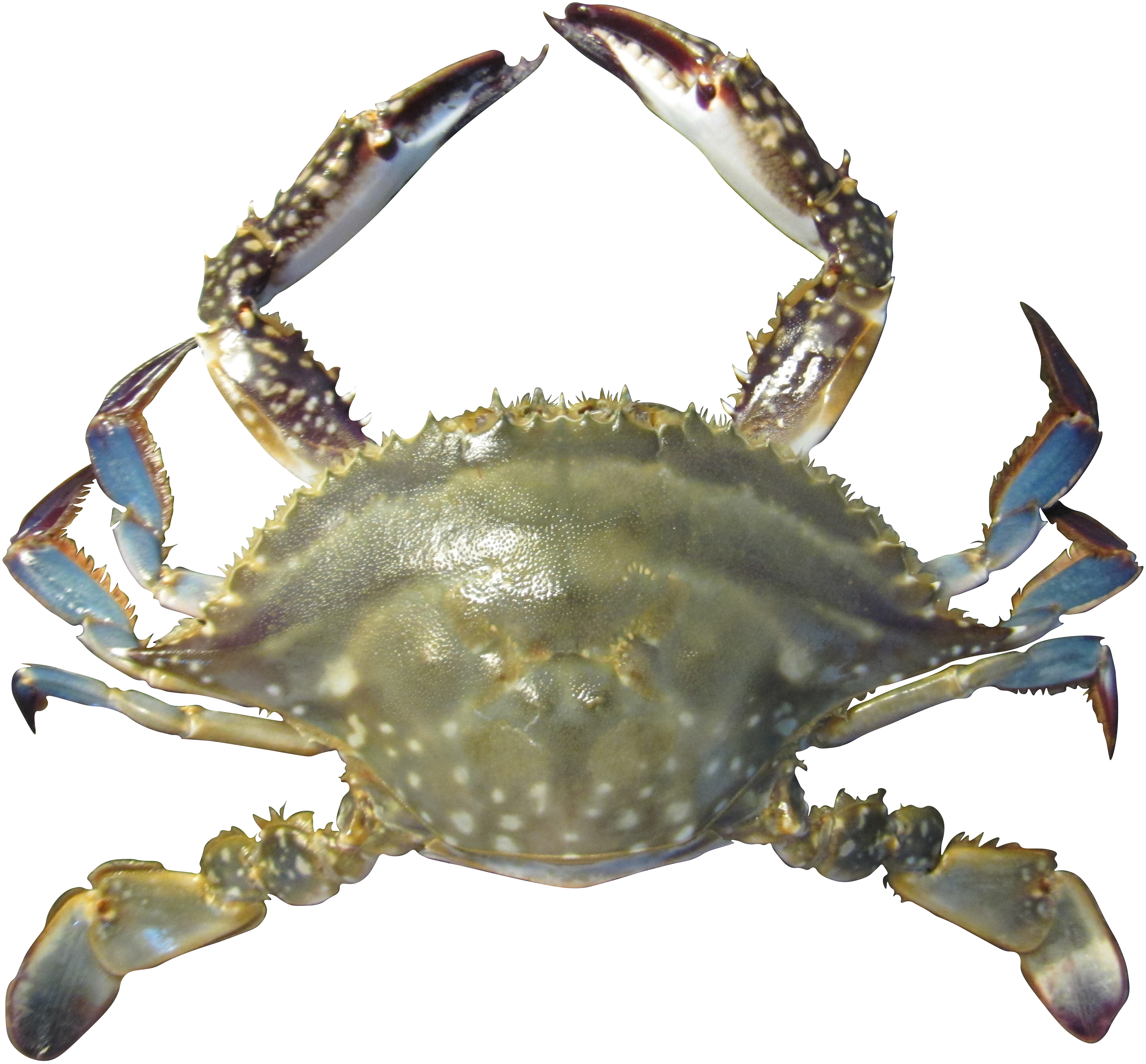

Click here to access/download;Figure;Figure 2.pdf

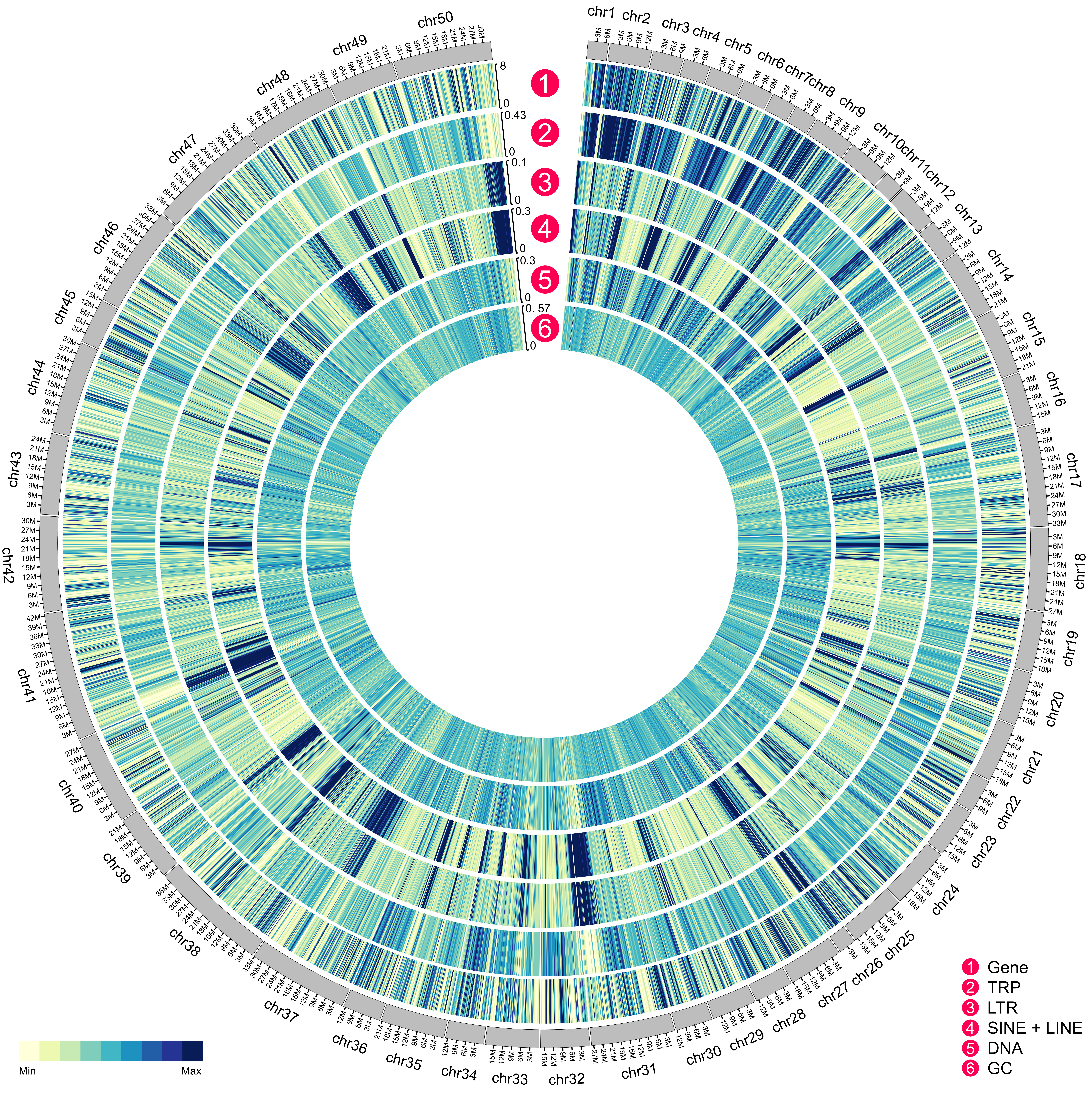

Click here to access/download;Figure;Figure 3.pdf

Figure 3

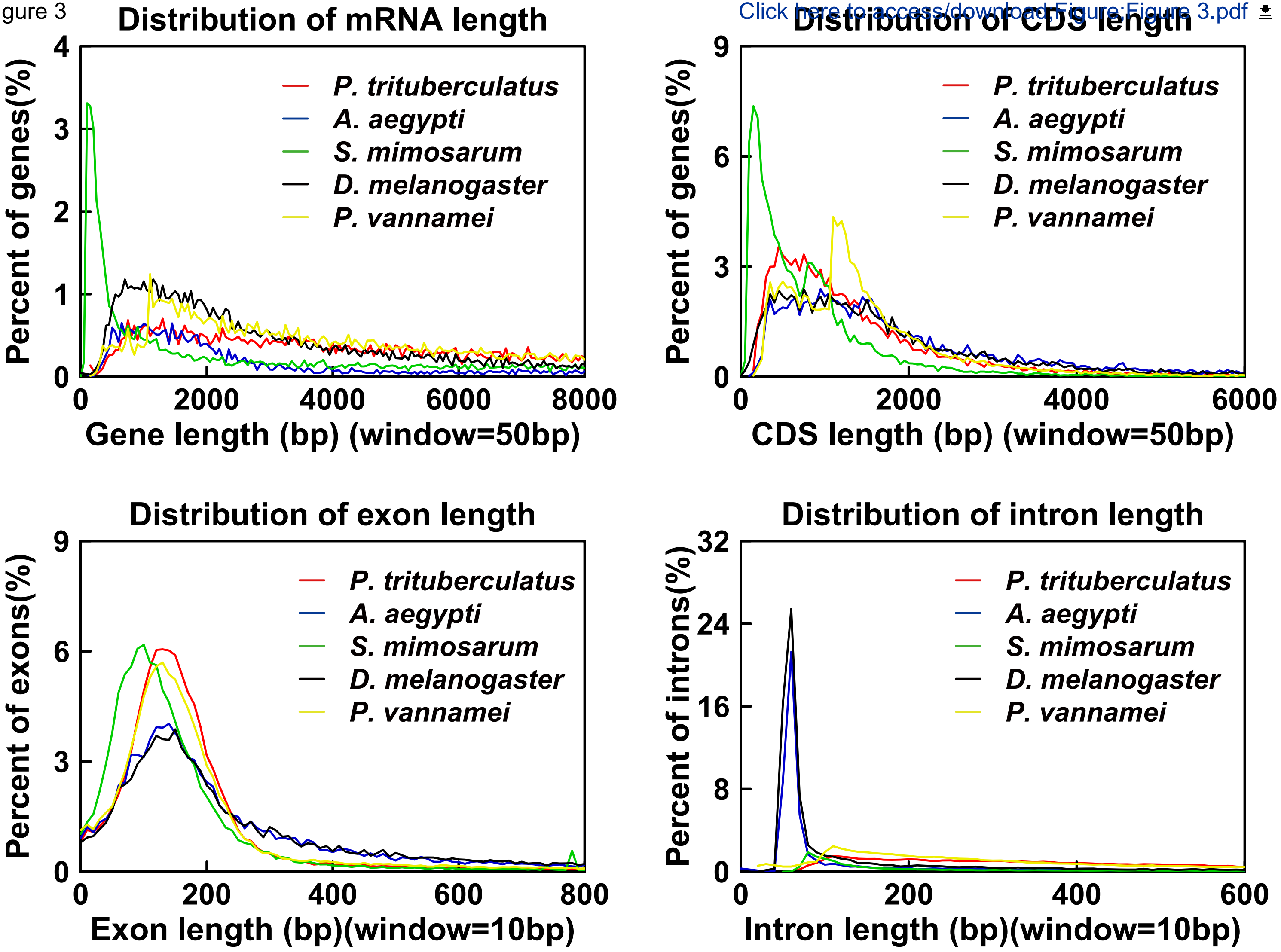

Figure 4

Click here to access/download;Figure;Figure 4.pdf

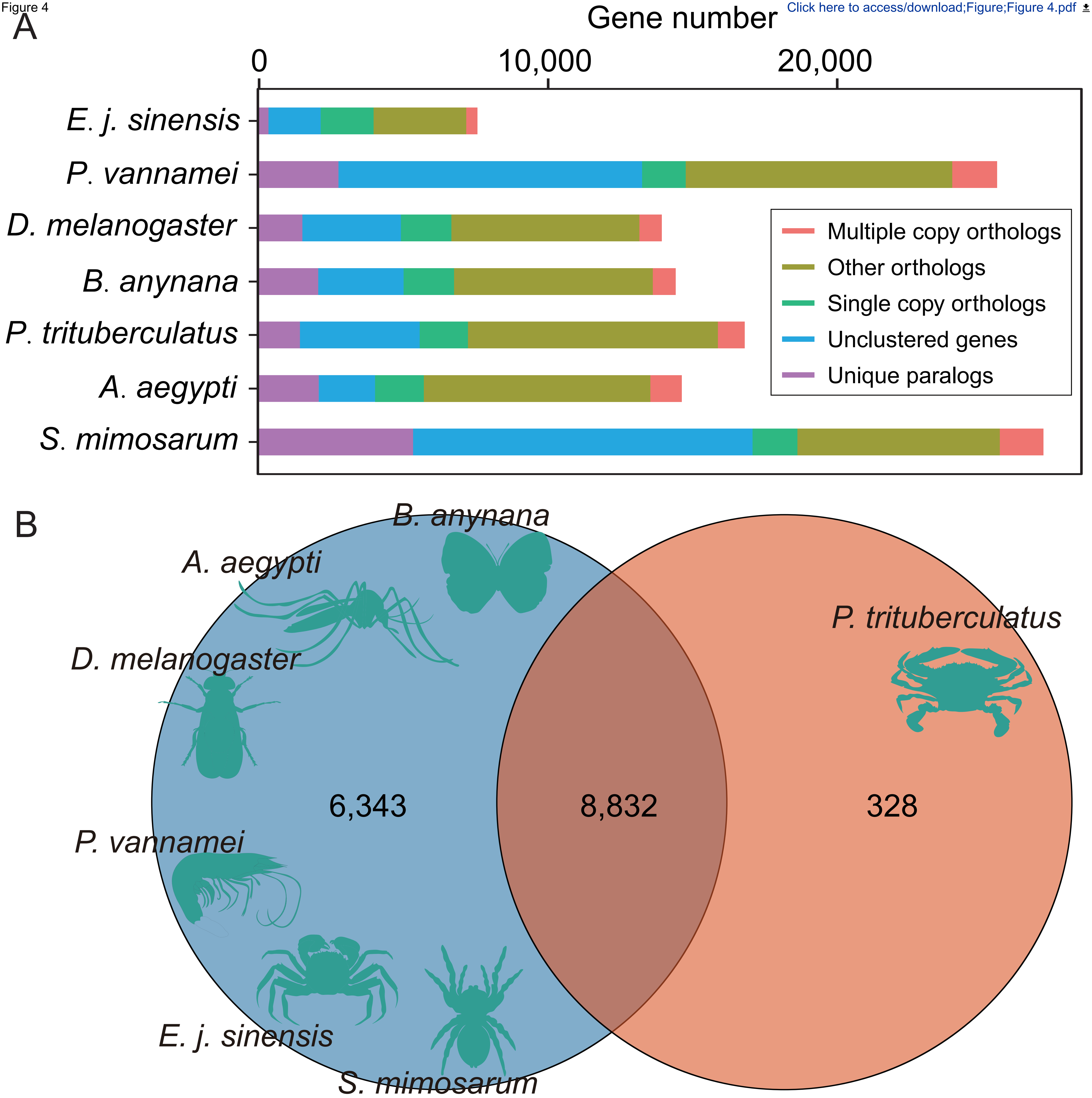

Figure 5

Click here to access/download;Figure;Figure 5.pdf

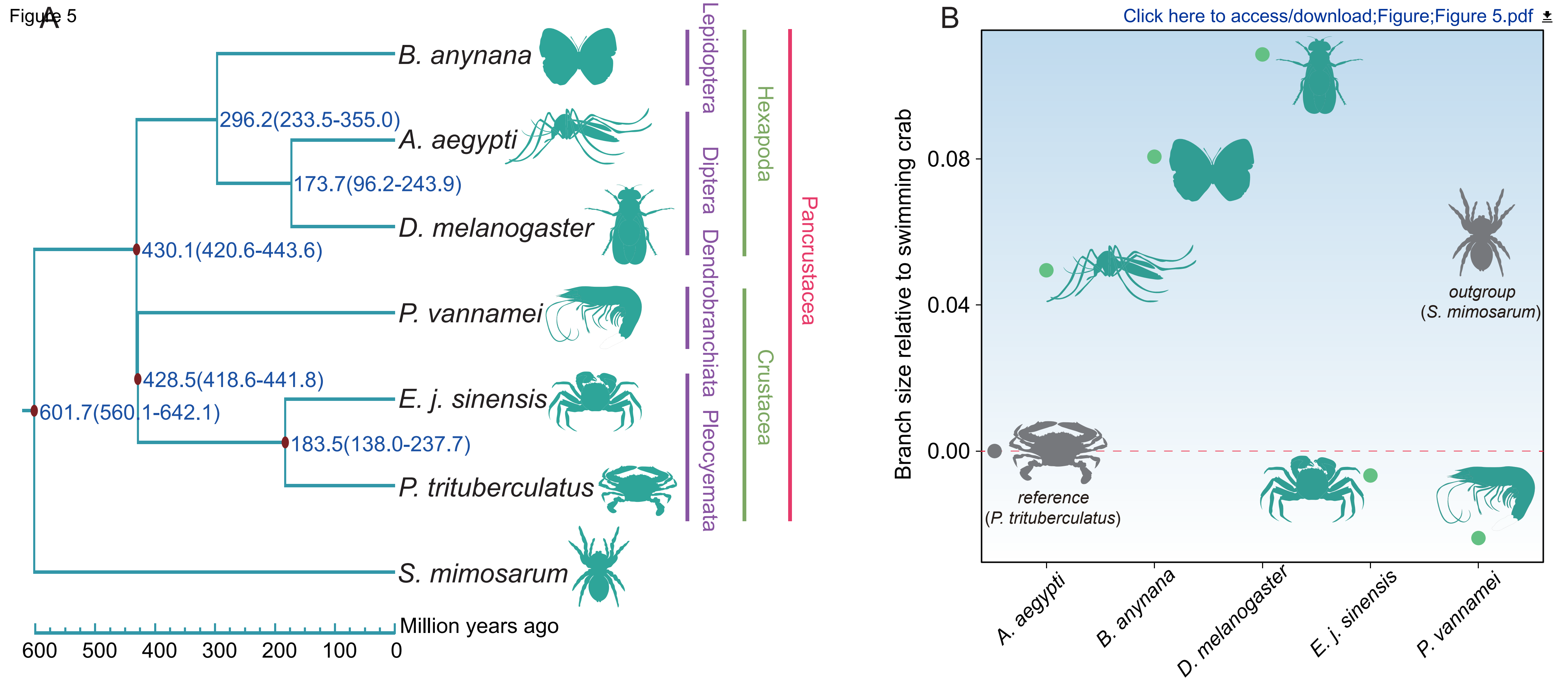

Click here to access/download
Supplementary Material
Supplementary tables and figures_20191112.docx
