# Supplementary material for: Chromosome-level genome assembly reveals the unique genome evolution of the swimming crab (Portunus trituberculatus)
Source: Gigascience. 2020 Jan 6;9(1):giz161. doi: 10.1093/gigascience/giz161 (PMC6944217; doi:10.1093/gigascience/giz161)

**Table S1: Statistics on genome sequencing data from BGISEQ platform.** These data are produced by short insert library with insert size 300bp, and the results were shown by the filtered reads. The sequencing depth was calculated by the assembled genome size.

| Term | Read pairs | Total bases (bp) | Sequencing strategy | Sequencing depth(X) |
| --- | --- | --- | --- | --- |
| PE-1 | 1,027,526,394 | 102,701,386,698 | PE100 | 102.29 |
| PE-2 | 1,027,526,394 | 102,701,386,698 | PE100 | 102.29 |
| Total | 2,055,052,788 | 205,402,773,396 | - | 204.58 |

**Table S2: Statistics on sequencing reads from Oxford Nanopore platform.** The reads with quality value Q > 7 and length > 1000 bp were considered.

| Lib-ID | Average length (bp) | N50 length (bp) | Reads number | Total base(bp) |
| --- | --- | --- | --- | --- |
| 20181001-NPL0319-P1-A9-D9 | 16,534 | 20,019 | 664,205 | 10,982,387,114 |
| 20181121-NPL0319-P1-A9-D9 | 18,690 | 22,467 | 901,917 | 16,857,057,292 |
| 20181125-NPL0319-P1-E7-H7 | 18,071 | 21,615 | 860,151 | 15,543,792,372 |
| 20181126-NPL0319-P2-A7-D7 | 18,294 | 22,227 | 633,280 | 11,585,843,315 |
| Total | - | - | 3,059,553 | 54,969,080,093 |

**Table S3: Statistics on Hi-C sequencing data.**

| Term | Read pairs (PE) | Total bases (PE) | Sequencing strategy | Sequencing depth (X) |
| --- | --- | --- | --- | --- |
| Hi-C library | 639,697,706 | 95,954,655,900 | PE150 | 95.47 |

**Table S4: Statistics on assembled chromosome-level genome by 3D *de novo* assembly software.**

| Chromosome ID | Length (bp)/Percentage (%) | Chromosome ID | Length (bp)/Percentage (%) |
| --- | --- | --- | --- |
| Hic_chr_1 | 6,801,519 | Hic_chr_26 | 5,847,757 |
| Hic_chr_2 | 13,909,200 | Hic_chr_27 | 18,172,300 |
| Hic_chr_3 | 10,158,951 | Hic_chr_28 | 12,784,577 |
| Hic_chr_4 | 8,581,979 | Hic_chr_29 | 14,785,251 |
| Hic_chr_5 | 11,256,687 | Hic_chr_30 | 12,291,475 |
| Hic_chr_6 | 9,867,398 | Hic_chr_31 | 27,702,984 |
| Hic_chr_7 | 8,085,266 | Hic_chr_32 | 16,283,586 |
| Hic_chr_8 | 7,550,507 | Hic_chr_33 | 17,720,624 |
| Hic_chr_9 | 13,971,342 | Hic_chr_34 | 12,697,713 |
| Hic_chr_10 | 14,151,211 | Hic_chr_35 | 21,343,038 |
| Hic_chr_11 | 6,730,153 | Hic_chr_36 | 12,712,161 |
| Hic_chr_12 | 14,164,360 | Hic_chr_37 | 33,242,319 |
| Hic_chr_13 | 12,285,065 | Hic_chr_38 | 38,839,762 |
| Hic_chr_14 | 21,622,326 | Hic_chr_39 | 22,338,078 |
| Hic_chr_15 | 21,825,099 | Hic_chr_40 | 29,382,946 |
| Hic_chr_16 | 16,942,622 | Hic_chr_41 | 42,710,960 |
| Hic_chr_17 | 33,887,922 | Hic_chr_42 | 31,792,048 |
| Hic_chr_18 | 27,450,777 | Hic_chr_43 | 26,812,371 |
| Hic_chr_19 | 19,786,189 | Hic_chr_44 | 30,753,393 |
| Hic_chr_20 | 17,756,556 | Hic_chr_45 | 15,010,712 |
| Hic_chr_21 | 19,494,137 | Hic_chr_46 | 33,906,160 |
| Hic_chr_22 | 11,273,125 | Hic_chr_47 | 38,170,672 |
| Hic_chr_23 | 16,378,840 | Hic_chr_48 | 30,197,846 |
| Hic_chr_24 | 20,371,438 | Hic_chr_49 | 21,793,880 |
| Hic_chr_25 | 18,751,408 | Hic_chr_50 | 32,585,247 |
| Chromosome length | | 982,931,937 | |
| Total length | | 1,005,046,021 | |
| Chromosome/total | | 97.80% | |

**Table S5. The quality comparison of these two genomes.**

| Term | Our genome assembly | Lv et al., 2017. |
| --- | --- | --- |
| Genome size | 1,005,046,021 bp | 842,129,340 bp |
| Contig N50 | 4,109,061 bp | 756 bp |
| Scaffold N50 | 21,793,880 bp | 1,154 bp |
| Number of contigs | 2,450 | 1,268,724 |
| Number of scaffolds | 523 | 898,300 |

**Table S6. The mapping results of genomic markers to the assembled genome.**

| Scaffold/Chromosome name | Length | Marker number | Scaffold/chromosome name | Length | Marker number |
| --- | --- | --- | --- | --- | --- |
| chromosome 1 | 6,801,519 | 57 | chromosome 50 | 32,585,247 | 311 |
| chromosome 2 | 13,909,200 | 70 | scaffold 64 | 350,985 | 5 |
| chromosome 3 | 10,158,951 | 72 | scaffold 67 | 338,168 | 3 |
| chromosome 4 | 8,581,979 | 94 | scaffold 71 | 73,508 | 2 |
| chromosome 5 | 11,256,687 | 129 | scaffold 147 | 4,323 | 1 |
| chromosome 6 | 9,867,398 | 82 | scaffold 202 | 962,959 | 4 |
| chromosome 7 | 8,085,266 | 65 | scaffold 203 | 482,353 | 2 |
| chromosome 8 | 7,550,507 | 54 | scaffold 204 | 355,038 | 1 |
| chromosome 9 | 13,971,342 | 149 | scaffold 205 | 1,045,770 | 13 |
| chromosome 10 | 14,151,211 | 132 | scaffold 211 | 67,536 | 1 |
| chromosome 11 | 6,730,153 | 13 | scaffold 212 | 76,608 | 1 |
| chromosome 12 | 14,164,360 | 170 | scaffold 214 | 66,484 | 1 |
| chromosome 13 | 12,285,065 | 147 | scaffold 218 | 108,407 | 2 |
| chromosome 14 | 21,622,326 | 219 | scaffold 219 | 129,740 | 1 |
| chromosome 15 | 21,825,099 | 202 | scaffold 221 | 155,387 | 2 |
| chromosome 16 | 16,942,622 | 279 | scaffold 222 | 73,640 | 1 |
| chromosome 17 | 33,887,922 | 464 | scaffold 223 | 158,863 | 1 |
| chromosome 18 | 27,450,777 | 274 | scaffold 224 | 96,760 | 1 |
| chromosome 19 | 19,786,189 | 250 | scaffold 225 | 182,313 | 2 |
| chromosome 20 | 17,756,556 | 252 | scaffold 227 | 320,803 | 2 |
| chromosome 21 | 19,494,137 | 184 | scaffold 229 | 52,492 | 1 |
| chromosome 22 | 11,273,125 | 55 | scaffold 255 | 14,546 | 1 |
| chromosome 23 | 16,378,840 | 235 | scaffold 267 | 36,773 | 2 |
| chromosome 24 | 20,371,438 | 253 | scaffold 286 | 216,572 | 1 |
| chromosome 25 | 18,751,408 | 209 | scaffold 296 | 72,252 | 1 |
| chromosome 26 | 5,847,757 | 37 | scaffold 301 | 15,188 | 2 |
| chromosome 27 | 18,172,300 | 199 | scaffold 314 | 179,449 | 2 |
| chromosome 28 | 12,784,577 | 184 | scaffold 321 | 23,948 | 1 |
| chromosome 29 | 14,785,251 | 184 | scaffold 338 | 14,669 | 1 |
| chromosome 30 | 12,291,475 | 122 | scaffold 371 | 42,619 | 1 |
| chromosome 31 | 27,702,984 | 280 | scaffold 381 | 14,542 | 1 |
| chromosome 32 | 16,283,586 | 167 | scaffold 393 | 557,757 | 9 |
| chromosome 33 | 17,720,624 | 160 | scaffold 394 | 241,797 | 1 |
| chromosome 34 | 12,697,713 | 77 | scaffold 395 | 335,263 | 2 |
| chromosome 35 | 21,343,038 | 192 | scaffold 396 | 748,793 | 5 |
| chromosome 36 | 12,712,161 | 133 | scaffold 408 | 199,450 | 4 |
| chromosome 37 | 33,242,319 | 404 | scaffold 410 | 386,818 | 2 |
| chromosome 38 | 38,839,762 | 451 | scaffold 420 | 85,288 | 1 |
| chromosome 39 | 22,338,078 | 209 | scaffold 444 | 5,319 | 1 |
| chromosome 40 | 29,382,946 | 437 | scaffold 445 | 15,009 | 1 |
| chromosome 41 | 42,710,960 | 274 | scaffold 448 | 240,817 | 1 |
| chromosome 42 | 31,792,048 | 407 | scaffold 452 | 1,072,529 | 10 |
| chromosome 43 | 26,812,371 | 261 | scaffold 453 | 265,712 | 1 |
| chromosome 44 | 30,753,393 | 372 | scaffold 504 | 18,580 | 1 |
| chromosome 45 | 15,010,712 | 177 | scaffold 516 | 110,253 | 1 |
| chromosome 46 | 33,906,160 | 435 | scaffold 519 | 116,047 | 1 |
| chromosome 47 | 38,170,672 | 453 | scaffold 521 | 14,960 | 1 |
| chromosome 48 | 30,197,846 | 390 | scaffold 522 | 968,829 | 9 |
| chromosome 49 | 21,793,880 | 343 | scaffold 523 | 663,829 | 17 |

**Table S7: Statistics on mapping ratio of the BGISEQ short reads to swimming crab genome.**

| # Total Reads | # Mapped Reads | Mapped Ratio (%) | # PE mapped Reads | PE mapped Ratio (%) | # SE mapped Reads | SE mapped Ratio (%) |
| --- | --- | --- | --- | --- | --- | --- |
| 2,062,764,986 | 2,038,692,007 | 98.83 | 1,969,810,534 | 95.85 | 4,272,705 | 0.21 |

Note: PE mapped represent reads being mapped to the genome as read pairs, SE mapped represents reads being mapped to the genome as single reads.

**Table S8: Statistics on RNA-seq data.** These data are produced by short insert library with insert size 300bp, and the results were shown by the filtered reads.

| Term | Insert size (bp) | Read pairs (PE) | Total bases (PE) | Sequencing strategy |
| --- | --- | --- | --- | --- |
| RNA-seq library | 300 | 36,940,472 | 5,171,666,080 | PE150 |

**Table S9: Statistics on assembled transcripts by Bridger software.**

| Term | Size (bp) | Number |
| --- | --- | --- |
| N90 | 390 | 41,448 |
| N80 | 715 | 27,428 |
| N70 | 1,140 | 19,249 |
| N60 | 1,618 | 13,823 |
| N50 | 2,124 | 9,844 |
| Max length (bp) | 21,130 | - |
| Total length (bp) | 73,840,050 | - |
| Total number (>100 bp) | - | 68,685 |
| Total number (>10 kb) | - | 111 |

**Table S10: Statistics on transcript mapping ratio of swimming crab genome.**

| Mapped transcripts number | Total transcripts number | Mapping ratio |
| --- | --- | --- |
| 69,731 | 71,306 | 97.80% |

**Table S11: Genome quality comparison of swimming crab with other species.**

| Species | Genome source | Assembly | Genome size | N50 | Total  scaffold | BUSCO |
| --- | --- | --- | --- | --- | --- | --- |
| *Drosophila melanogaster* | NCBI | Chromosome | 143,726,002 | 25,286,936 | 1,870 | 99.7% |
| *Mus musculus* | NCBI | Chromosome | 2,818,974,548 | 130,694,993 | 239 | 83.2% |
| *Bombus terrestris* | NCBI | Chromosome | 248,654,244 | 12,868,931 | 5,609 | 96.7% |
| *Mesobuthus martensii* | NCBI | Contig | 925,546,267 | 45,228 | 92,408 | 58.4% |
| *Stegodyphus mimosarum* | NCBI | Scaffold | 2,738,704,917 | 480,636 | 68,653 | 88.5% |
| *Aedes aegypti* | NCBI | Chromosome | 1,870,673,364 | 310,827,022 | 6,534 | 99.4% |
| *Penaeus vannamei* | NCBI | Scaffold | 1,663,565,311 | 605,555 | 4,682 | 90.4% |
| *Bicyclus anynana* | NCBI | Scaffold | 475,399,557 | 638,282 | 10,800 | 97.7% |

**Table S12: Statistics on annotated repetitive sequences using different software.**

| Type | Repeat Size | % of genome |
| --- | --- | --- |
| Trf | 193,555,524 | 19.28 |
| Repeatmasker | 213,558,503 | 21.27 |
| Proteinmask | 88,375,336 | 8.8 |
| *De novo* | 464,908,824 | 46.3 |
| Total | 547,392,656 | 54.52 |

**Table S13: KEGG enrichment analysis of unique gene families in swimming crab relative to six other species.**

| Map number | Pathway | Count | *P*-value | *Q*-value |
| --- | --- | --- | --- | --- |
| map04730 | Long-term depression | 21 | 4.82E-06 | 0.000111246 |
| map04141 | Protein processing in endoplasmic reticulum | 27 | 4.84E-06 | 0.000111246 |
| map05219 | Bladder cancer | 13 | 8.03E-06 | 0.000164109 |
| map05150 | Staphylococcus aureus infection | 8 | 1.00E-05 | 0.000184164 |
| map05223 | Non-small cell lung cancer | 15 | 1.20E-05 | 0.000201043 |
| map05218 | Melanoma | 13 | 1.74E-05 | 0.000266794 |
| map04973 | Carbohydrate digestion and absorption | 15 | 1.94E-05 | 0.000273928 |
| map04650 | Natural killer cell mediated cytotoxicity | 15 | 3.04E-05 | 0.000399532 |
| map05033 | Nicotine addiction | 12 | 6.02E-05 | 0.000738549 |
| map05213 | Endometrial cancer | 13 | 6.74E-05 | 0.000775245 |
| map05221 | Acute myeloid leukemia | 13 | 7.86E-05 | 0.000850901 |
| map05214 | Glioma | 15 | 9.10E-05 | 0.000930322 |
| map03018 | RNA degradation | 16 | 9.71E-05 | 0.00094032 |
| map05212 | Pancreatic cancer | 13 | 0.000185892 | 0.001710206 |
| map00052 | Galactose metabolism | 15 | 0.00021267 | 0.001863395 |
| map04724 | Glutamatergic synapse | 18 | 0.000223081 | 0.00186577 |
| map04012 | ErbB signaling pathway | 15 | 0.000266574 | 0.002132592 |
| map05215 | Prostate cancer | 13 | 0.000312935 | 0.002399169 |
| map05210 | Colorectal cancer | 13 | 0.000354338 | 0.002607928 |
| map04330 | Notch signaling pathway | 9 | 0.000505743 | 0.003579101 |
| map05211 | Renal cell carcinoma | 13 | 0.000796386 | 0.005427221 |
| map05220 | Chronic myeloid leukemia | 13 | 0.001094316 | 0.007191219 |
| map04080 | Neuroactive ligand-receptor interaction | 34 | 0.001355472 | 0.008600239 |
| map04720 | Long-term potentiation | 20 | 0.001681795 | 0.010315006 |
| map00053 | Ascorbate and aldarate metabolism | 8 | 0.001978638 | 0.011744174 |
| map04726 | Serotonergic synapse | 14 | 0.002811352 | 0.016165273 |
| map00040 | Pentose and glucuronate interconversions | 8 | 0.003498373 | 0.019506082 |
| map05160 | Hepatitis C | 13 | 0.003661202 | 0.019813562 |
| map04270 | Vascular smooth muscle contraction | 17 | 0.004895707 | 0.025737431 |
| map00830 | Retinol metabolism | 8 | 0.006518962 | 0.033319138 |
| map04068 | FoxO signaling pathway | 13 | 0.006861204 | 0.034120583 |
| map00982 | Drug metabolism - cytochrome P450 | 7 | 0.008029452 | 0.038879451 |
| map04150 | mTOR signaling pathway | 16 | 0.009805838 | 0.046263441 |
| map00860 | Porphyrin and chlorophyll metabolism | 7 | 0.010239689 | 0.047102568 |

**Table S14: Two-cluster analysis of swimming crab and other species.** This analysis was employed by LINTRE. Delta = | bA - bB |; Z = delta/s.e. (delta/standard error); CP (confident probability) = 1 – *P*-value.

| Outgroup | Ingroup1 | Ingroup2 | bA | bB | delta | s.e. | Z | CP |
| --- | --- | --- | --- | --- | --- | --- | --- | --- |
| *S. mimosarum* | *E. sinensis* | *P. trituberculatus* | 0.080882 | 0.087573 | 0.006691 | 0.001561 | 4.286421 | 99.96% |
| *S. mimosarum* | *P. vannamei* | *P. trituberculatus* | 0.141771 | 0.165606 | 0.023835 | 0.002025 | 11.769478 | 99.96% |
| *S. mimosarum* | *D. melanogaster* | *P. trituberculatus* | 0.559740 | 0.451069 | 0.108672 | 0.003316 | 32.775032 | 99.96% |
| *S. mimosarum* | *B. anynana* | *P. trituberculatus* | 0.527380 | 0.446749 | 0.080631 | 0.003244 | 24.852662 | 99.96% |
| *S. mimosarum* | *A. aegypti* | *P. trituberculatus* | 0.502437 | 0.452533 | 0.049904 | 0.003163 | 15.778682 | 99.96% |

**Table S15: KEGG enrichment analysis of expanded gene families in swimming crab.**

| Map number | Pathway | Count | *P*-value | *Q*-value |
| --- | --- | --- | --- | --- |
| map04066 | HIF-1 signaling pathway | 24 | 2.62E-05 | 0.000109025 |
| map04510 | Focal adhesion | 39 | 3.34E-05 | 0.000135977 |
| map04390 | Hippo signaling pathway | 21 | 4.64E-05 | 0.000184649 |
| map00053 | Ascorbate and aldarate metabolism | 12 | 4.78E-05 | 0.000185981 |
| map01200 | Carbon metabolism | 24 | 7.01E-05 | 0.000266791 |
| map04919 | Thyroid hormone signaling pathway | 22 | 7.39E-05 | 0.000275444 |
| map00830 | Retinol metabolism | 13 | 7.82E-05 | 0.000285669 |
| map04910 | Insulin signaling pathway | 29 | 9.99E-05 | 0.000357592 |
| map04015 | Rap1 signaling pathway | 35 | 0.00011955 | 0.000419596 |
| map00520 | Amino sugar and nucleotide sugar metabolism | 21 | 0.000212121 | 0.000730187 |
| map04974 | Protein digestion and absorption | 23 | 0.000237762 | 0.000803009 |
| map04614 | Renin-angiotensin system | 7 | 0.000243106 | 0.000805851 |
| map00140 | Steroid hormone biosynthesis | 13 | 0.000251114 | 0.000817261 |
| map04916 | Melanogenesis | 18 | 0.000353068 | 0.001118369 |
| map05204 | Chemical carcinogenesis | 13 | 0.000356129 | 0.001118369 |
| map03020 | RNA polymerase | 15 | 0.000362956 | 0.001120159 |
| map00603 | Glycosphingolipid biosynthesis - globo series | 8 | 0.000482319 | 0.001463306 |
| map00601 | Glycosphingolipid biosynthesis - lacto and neolacto series | 9 | 0.000684455 | 0.002041956 |
| map04921 | Oxytocin signaling pathway | 23 | 0.000928741 | 0.002725323 |
| map00982 | Drug metabolism - cytochrome P450 | 9 | 0.003821397 | 0.011032743 |
| map00860 | Porphyrin and chlorophyll metabolism | 9 | 0.005215886 | 0.014819741 |
| map00040 | Pentose and glucuronate interconversions | 9 | 0.00697535 | 0.01920904 |
| map00980 | Metabolism of xenobiotics by cytochrome P450 | 9 | 0.00697535 | 0.01920904 |
| map00983 | Drug metabolism - other enzymes | 9 | 0.01684035 | 0.045673069 |

**Figure S1: 17-mer analysis of swimming crab genome.**


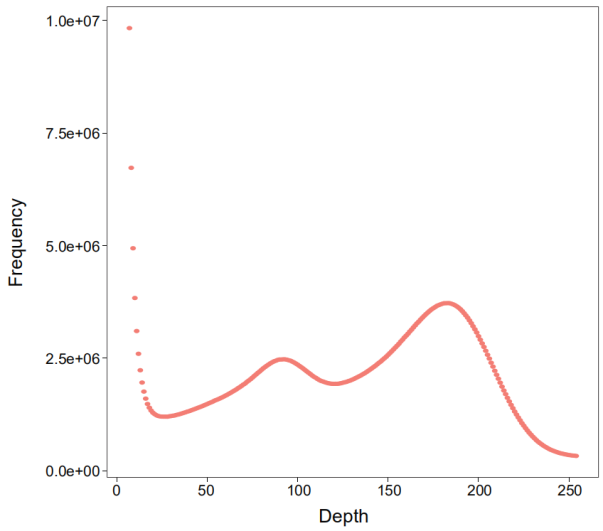


**Figure S2: GC distribution in species.**


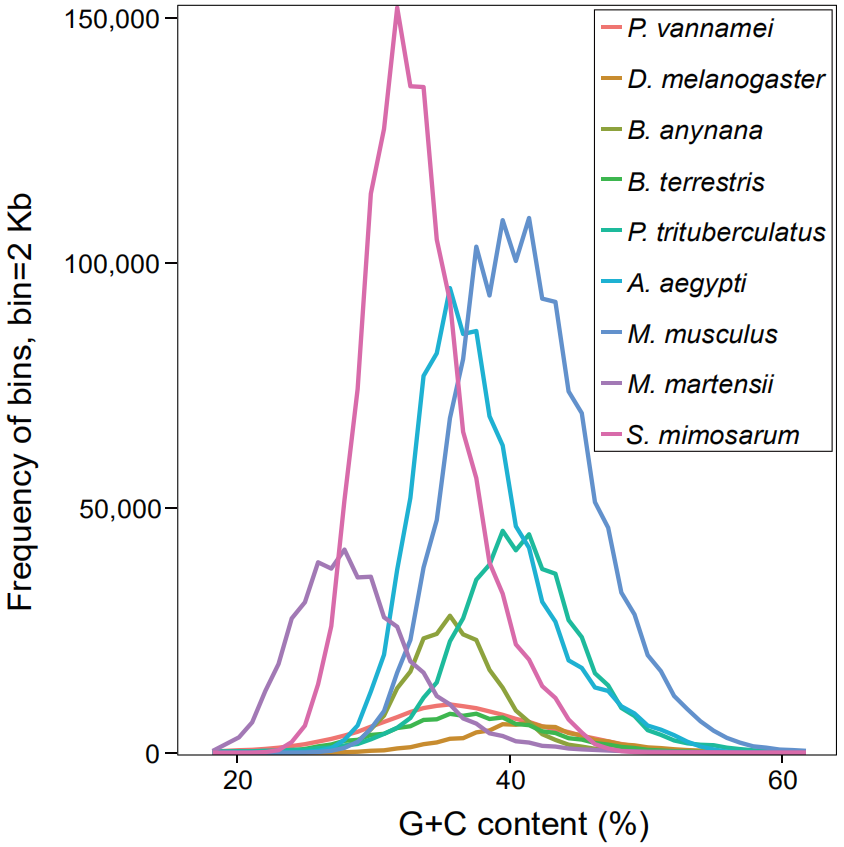

Supplement: giz161_Supplemental_Tables_and_Figures [file giz161_supplemental_tables_and_figures.docx]
