# Supplementary material for: Chromosome-level genome assembly reveals the unique genome evolution of the swimming crab (Portunus trituberculatus)
Source: Gigascience. 2020 Jan 6;9(1):giz161. doi: 10.1093/gigascience/giz161 (PMC6944217; doi:10.1093/gigascience/giz161)
Supplement: giz161_Response_to_Reviewer_Comments_Original_Submission [file giz161_response_to_reviewer_comments_original_submission.pdf]

Author's Response To Reviewer Comments

Close

Reviewer #1 Comments for the Author…
1. The authors need to mention a previous publication describing the genome of P. tuberculanus: Lv J, Gao B, Liu P, Li J, Meng X. Linkage mapping aided by de novo genome and transcriptome assembly in Portunus trituberculatus: applications in growth-related QTL and gene identification. Sci Rep. 2017;7: 7874. doi:10.1038/s41598-017-08256-8). They need to compare their results with that of Lv et al., explain how they add to this previous knowledge, and discuss the discrepancies. The sentence at lines 70-71 should also be corrected, as it is not accurate that 'genomic research on the swimming crab has only been conducted at the transcriptome level, with the whole genome not yet described'.
Response: We appreciate this reviewer for the comments and suggestions. We have cited the paper (Lv et al., 2017) and corrected the sentence at lines 70-71 in the revised manuscript. Moreover, we also compared the published paper of Lv et al and the genome we assembled, and the results indicated that our genome assembly is obviously better than the published paper (Lv et al., 2017). This results were added in the revised supplementary files (Table S5). Thank you.

2. The manuscript identifies 50 chromosomes in P. tuberculanus genome, while linkage analysis of genomic markers lead Lv and colleagues to describe 53 linkage groups. The authors should discuss this discrepancy. In particular, it would be informative to map the 10000 markers used by Lv and colleagues to the proposed chromosomal assembly and compare to the genetic map.
Response: We appreciate this reviewer for the comments and suggestions. Using the 10,963 markers in Lv et al., 2017 paper, all these markers were mapped to our genome using blastn with e-value of 10-5. Then, we found that 10,897 markers (99.40%) can be found in our genome, which proved that our genome have high-quality and completeness. What's more, the 50 assembled chromosomes have 10,769 markers, which accounts for almost 98.83% of all the mapped markers. The other scaffolds only have ~1.17% markers, and only three scaffolds (scaffold 205, scaffold 452, and scaffold 523) have more than 10 markers (including 10). Moreover, the length of these three scaffolds is quite short, which indicates that these three scaffolds may be part of some chromosomes, not a complete chromosome. So we think that the swimming crab genome should have 50 chromosomes. This table was added in the revised supplementary files (Table S6). Thank you.

3. Genome size estimation is performed on the sole basis of the position of maximal density of 17-mers in BGISeq-500 short reads. However, the k-mer curve presented in Figure S1 has an unusually flat shape, with a secondary peak at ~90 depth, which casts some doubt on the accuracy of this measure. Moreover, the proposed estimation (1 Gb) differs substantially from that made by Lv and colleagues, who put forward a size of 0.8 Gb based on the analysis of 23-mer frequency. Given the importance of this result for estimating the degree of completion of the proposed assembly, the authors need to provide a more solid genome size estimation, either by choosing an alternative method (eg flow cytometry), providing k-mer counts for higher k, or otherwise explaining both the unusual shape of their k-mer curve and the discrepancy between their estimation and the one in Lv et al., 2017.
Response: We appreciate this reviewer for the comments and suggestions. We also used k-mer frequency with k set as 23 to check the genome size this time, and the estimated genome size is 959,508,443 bp, which is very close with the assembled genome size. Due to the high rate of heterozygosity, Figure S1 has two peaks and the shape looks a little flat. But the accuracy of this measure is reliable, and the peak of heterozygosity (80) is exactly 1/2 of the main peak (160), indicating the estimated results is reliable.

4. The submitted manuscript lacks all figure legends. Although this is probably due to a mistake during the submission process, this makes the signification of some figures very difficult to understand. In particular Figure 4a is of no use as long as what is described as 'unclustered genes', 'unique paralogues', 'multiple copy orthologs' or 'other orthologs' is not defined. A revised submission must of course include descriptive figure legends.
Response: Sorry for this mistake, we have added the figure legends in the revised manuscript. Thank you.

5. It would be of interest to report the rate of heterozygosity found in the sequenced individual.
Response: The rate of heterozygosity in the sequenced swimming crab individual was calculated using k-mer and the rate is ~0.9%.

6. l 168-170 : the method for gene annotation using BLAST similarity with KEGG, SwissProt and TrEMBL databases should be more detailed : which BLAST program ? which parameter values ? which subject species ? which score threshold required to call an annotation ? Etc.
Response: Thank you for your suggestions. We have added the information you mentioned in the revised manuscript. Thank you.

7. l 99-100 : the sentence 'For the short reads, any reads with more than 10% unknown reads or 100 low-quality bases more than 50% along with its paired-end read were removed' is very obscure. Please clarify.
Response: Sorry for this misleading. We have corrected this in the revised manuscript.

8. l 216 : please define precisely the seldom-used term 'mounting rate'.
Response: The mounting rate means the total length of the contigs that anchored to chromosomes divided by the total length of all assembled contigs. We also add the description of 'mounting rate' in the revised manuscript.

9. l 268-273 : picking 3 'interesting' terms out of the list of 34 KEGG terms that show enrichment in the unique gene families is very little informative and even misleading, especially when these 3 terms appear rather far (ranks 20, 29 and 30) in this list. This paragraph, and possibly the enrichment analysis itself should be discarded : the genome assembly and annotation are interesting enough in their own respect without having to add such poorly supported speculations.
Response: Sorry for this misleading. We have removed this analysis (enrichment analysis) in the revised manuscript. Thank you.

10. l 292-300 : The conclusions on the relative evolution rates are not appropriate. First, the authors should not use the ill-defined term 'survival pressure'. Second, it is oversimplistic to interpret faster or slower evolution rates in terms of selection, since these rates are influenced by many other factors, including mutation rates, population size, etc.
Response: Thank you for your suggestions. We have revised the words in this part (including 'survival pressure') in the revised manuscript.

11. The authors should remove any reference to 'adaptive evolution' from the manuscript title, since nothing in their data points to evidence of adaptive evolution.

Response: We have removed the words of 'adaptive evolution' in manuscript title in the revised manuscript. Thank you.

12. l 294 and 191-194: Please explain the choice of LINTRE, a rather uncommon tool in the field, to assess evolution rates. Please also provide more precise reference (the cited 1995 paper does not directly refer to a program), program version, choice of parameters.
Response: Thank you for your suggestions. LINTRE is a pretty old software but it is very useful, which also used in some other papers, such as in the NATURE paper "The seahorse genome and the evolution of its specialized morphology" (Lin et al.) that published in 2016. We used the version 1 of LINTRE and all the parameters were used as default. We also make a change in our manuscript. Thank you.

13. L302-311: the last paragraph of the results is highly speculative, with no interpretation as to why the crab-enriched signalling pathways (HIF1, Hippo and insulin) might be particularly relevant to the evolution of this species. Although factually correct, this analysis could easily be removed from the manuscript to lend more weight to its important parts: the assembly and annotation of the genome.
Response: Thank you for your suggestions. We have removed this part in the revised manuscript.

14. L492: Reference 48 has no author names.
Response: Sorry for this mistake, we have corrected it in the revised manuscript.

15. Availability of the data: in the case of a revised submission, it would be more convenient for reviewers if they could have access to the genome data in the same form that will be eventually available from GigaDB. At present, nothing can be accessed there.
Response: We have uploaded all the related data, including the data of genome assembly and

annotation to GigaDB. Thank you.

Reviewer #2 Comments for the Author…
1. This paper describes a high-quality genome assembly of Portunus trituberculatus, one of the most widely fished species of crab in the world. The paper is written clearly and succinctly. The figures look excellent are clear and informative. I downloaded the genome and found an expected Hox cluster, which is in line with this being a high quality data set. The authors do not describe the Hox cluster and this is fine, but they should consider since it would not have to be that extensive of an analysis and a description plus Hox complex figure would increase the interest in the paper (it could also be a follow-up study). Nevertheless, this is a wonderful genomic resource, an excellent analysis, and if the authors address the reproducibility issues in my next paragraph, I would say that this is a model genome data note.
Response: Thank you for your suggestions. Yes, hox cluster annotation and hox gene evolution analysis are the next points in our follow-up study, and several crab species may be included. Thank you.

2. The methods appear thorough, however repeating these analyses in full would be impossible without guessing at some parameter settings etc. In order to make the work repeatable, please include ALL command lines in a supplemental document. There is an excellent example in the supplement linked here: https://academic.oup.com/mbe/article/35/2/486/4644721#113627427
Response: Thank you for your suggestions. In order to make all the parameter setting clear, we have added the parameter of the software in the revised manuscript refer to the applied example. Thank you.

3. Line 34: "only limited transcriptome data currently available"
--This is untrue as there is a draft genome assembly available in GenBank:
https://www.ncbi.nlm.nih.gov/nuccore/VSRR000000000.1
This available draft should be acknowledged in the manuscript (even though the assembly in this current study is far superior).
Response: Sorry for this mistake. We have corrected this description and added the comparison between our genome and the published genome. This table was added in the revised supplementary files (Table S5). Thank you.

4. Line 70: "genomic research on the swimming crab has only been conducted at the transcriptome level [14-16], with the whole genome not yet described. --Likewise, this line should be updated to mention this draft genome.
Response: Sorry for this mistake. We have corrected this mistake in the revised manuscript.

5. Line 84: "Muscle RNA was also extracted using TRIzol (Invitrogen) according to the manufacturer's instructions" -- Should clarify whether the same animal used was used for extraction of RNA asthe genome. Indeed it should also be noted if the same animal was used for all genomic sequencing.
Response: Thank you for your suggestions. The same animal was used in all the RNA and DNA sequencing. We have corrected this in the revised manuscript.

6. Line 297 (and line 300): "greater survival pressures on these two species"
--I wouldn't attribute faster evolutionary rates to "survival pressures." Evolutionary rate has more to do with generation time (shorter=greater) and population size (larger=greater). The evolutionary rate makes sense in relation to both of these factors. Differences in survival pressures are heavily influenced by competition in large populations.
Response: Thank you for your suggestions. We have modified the inappropriate words in the revised manuscript.

7. Table 2: It seems as if the "Summary" row represents the percentage for "Complete BUSCO (C)." The label "Summary" does not make sense in this context. I would rename to "Summary (percentage Complete Busco)" or "percentage Complete Busco"
Response: Thank you for your suggestions. We have corrected this mistake in the revised manuscript.

8. I love Figure 1! Beautiful creature!
Response: Thank you.

9. Figure 2 and 3 legends should include more information. For example, what program was used to generate figure. What is the underlying data from, etc.
Response: Thank you for your suggestions. We have added more information to describe these two figures in the revised manuscript. Thank you.

Close
