# Supplementary material for: Chromosome-level genome assembly reveals the unique genome evolution of the swimming crab (Portunus trituberculatus)
Source: Gigascience. 2020 Jan 6;9(1):giz161. doi: 10.1093/gigascience/giz161 (PMC6944217; doi:10.1093/gigascience/giz161)
Supplement: giz161_Reviewer_1_Report_Original_Submission — Hugues Roest Crollius -- 10/4/2019 Reviewed [file giz161_reviewer_1_report_original_submission.pdf]

Reviewer Report

Title: Chromosome-level genome assembly reveals the unique genome evolution of swimming crab (Portunus trituberculatus)

Version: Original Submission **Date:** 10/4/2019

Reviewer name: Hugues Roest Crollius

Reviewer Comments to Author:

The manuscript by Tang and colleagues presents the genome assembly of the swimming crab (Portunus trituberculatus) at the chromosome level. It is in itself a valuable contribution, especially considering the small number of available crustacean genomes. Quality controls of the presented assembly seem to meet current standards. There are however several important points that need to be addressed before publication can be considered.

Major points

The authors need to mention a previous publication describing the genome of P. tuberculanus: Lv J, Gao B, Liu P, Li J, Meng X. Linkage mapping aided by de novo genome and transcriptome assembly in Portunus trituberculatus: applications in growth-related QTL and gene identification. Sci Rep. 2017;7: 7874. doi:10.1038/s41598-017-08256-8).

They need to compare their results with that of Lv et al., explain how they add to this previous knowledge, and discuss the discrepancies. The sentence at lines 70-71 should also be corrected, as it is not accurate that 'genomic research on the swimming crab has only been conducted at the transcriptome level, with the whole genome not yet described'.

The manuscript identifies 50 chromosomes in P. tuberculanus genome, while linkage analysis of genomic markers lead Lv and colleagues to describe 53 linkage groups. The authors should discuss this discrepancy. In particular, it would be informative to map the 10000 markers used by Lv and colleagues to the proposed chromosomal assembly and compare to the genetic map.

Genome size estimation is performed on the sole basis of the position of maximal density of 17-mers in BGISeq-500 short reads. However, the k-mer curve presented in Figure S1 has an unusually flat shape, with a secondary peak at ~90 depth, which casts some doubt on the accuracy of this measure. Moreover, the proposed estimation (1 Gb) differs substantially from that made by Lv and colleagues, who put forward a size of 0.8 Gb based on the analysis of 23-mer frequency.

Given the importance of this result for estimating the degree of completion of the proposed assembly, the authors need to provide a more solid genome size estimation, either by choosing an alternative method (eg flow cytometry), providing k-mer counts for higher k, or otherwise explaining both the unusual shape of their k-mer curve and the discrepancy between their estimation and the one in Lv et al., 2017.

The submitted manuscript lacks all figure legends. Although this is probably due to a mistake during the submission process, this makes the signification of some figures very difficult to understand. In particular Figure 4a is of no use as long as what is described as 'unclustered genes', 'unique paralogues', 'multiple copy orthologs' or 'other orthologs' is not defined. A revised submission must of course include

descriptive figure legends.
Minor points :
It would be of interest to report the rate of heterozygosity found in the sequenced individual.
l 168-170 : the method for gene annotation using BLAST similarity with KEGG, SwissProt and TrEMBL databases should be more detailed : which BLAST program ? which parameter values ? which subject species ? which score threshold required to call an annotation ? Etc.
l 99-100 : the sentence 'For the short reads, any reads with more than 10% unknown reads or 100 low-quality bases more than 50% along with its paired-end read were removed' is very obscure. Please clarify.
l 216 : please define precisely the seldom-used term 'mounting rate'.
l 268-273 : picking 3 'interesting' terms out of the list of 34 KEGG terms that show enrichment in the unique gene families is very little informative and even misleading, especially when these 3 terms appear rather far (ranks 20, 29 and 30) in this list. This paragraph, and possibly the enrichment analysis itself should be discarded : the genome assembly and annotation are interesting enough in their own respect without having to add such poorly supported speculations.
l 292-300 : The conclusions on the relative evolution rates are not appropriate. First, the authors should not use the ill-defined term 'survival pressure'. Second, it is oversimplistic to interpret faster or slower evolution rates in terms of selection, since these rates are influenced by many other factors, including mutation rates, population size, etc.
The authors should remove any reference to 'adaptive evolution' from the manuscript title, since nothing in their data points to evidence of adaptive evolution.
l 294 and 191-194: Please explain the choice of LINTRE, a rather uncommon tool in the field, to assess evolution rates. Please also provide more precise reference (the cited 1995 paper does not directly refer to a program), program version, choice of parameters.
L302-311 : the last paragraph of the results is highly speculative, with no interpretation as to why the crab-enriched signalling pathways (HIF1, Hippo and insulin) might be particularly relevant to the evolution of this species. Although factually correct, this analysis could easily be removed from the manuscript to lend more weight to its important parts : the assembly and annotation of the genome.
l 492 : Reference 48 has no author names.
Availability of the data : in the case of a revised submission, it would be more convenient for reviewers if they could have access to the genome data in the same form that will be eventually available from GigaDB. At present, nothing can be accessed there.

Level of Interest

Please indicate how interesting you found the manuscript: Choose an item.

Quality of Written English

Please indicate the quality of language in the manuscript: Choose an item.

Declaration of Competing Interests

Please complete a declaration of competing interests, considering the following questions:

- Have you in the past five years received reimbursements, fees, funding, or salary from an organisation that may in any way gain or lose financially from the publication of this manuscript, either now or in the future?
- Do you hold any stocks or shares in an organisation that may in any way gain or lose financially from the publication of this manuscript, either now or in the future?
- Do you hold or are you currently applying for any patents relating to the content of the manuscript?
- Have you received reimbursements, fees, funding, or salary from an organization that holds or has applied for patents relating to the content of the manuscript?
- Do you have any other financial competing interests?
- Do you have any non-financial competing interests in relation to this paper?

If you can answer no to all of the above, write 'I declare that I have no competing interests' below. If your reply is yes to any, please give details below.

I declare that I have no competing interests

I agree to the open peer review policy of the journal. I understand that my name will be included on my report to the authors and, if the manuscript is accepted for publication, my named report including any attachments I upload will be posted on the website along with the authors' responses. I agree for my report to be made available under an Open Access Creative Commons CC-BY license (http://creativecommons.org/licenses/by/4.0/). I understand that any comments which I do not wish to be included in my named report can be included as confidential comments to the editors, which will not be published.

Choose an item.

To further support our reviewers, we have joined with Publons, where you can gain additional credit to further highlight your hard work (see: https://publons.com/journal/530/gigascience). On publication of this paper, your review will be automatically added to Publons, you can then choose whether or not to claim your Publons credit. I understand this statement.

Yes Choose an item.
