# Supplementary material for: Chromosome-level genome assembly reveals the unique genome evolution of the swimming crab (Portunus trituberculatus)
Source: Gigascience. 2020 Jan 6;9(1):giz161. doi: 10.1093/gigascience/giz161 (PMC6944217; doi:10.1093/gigascience/giz161)
Supplement: giz161_Reviewer_2_Report_Original_Submission — Joseph F. Ryan -- 10/4/2019 Reviewed [file giz161_reviewer_2_report_original_submission.pdf]

Reviewer Report

Title: Chromosome-level genome assembly reveals the unique genome evolution of swimming crab (Portunus trituberculatus)

Version: Original Submission **Date:** 10/4/2019

Reviewer name: Joseph F. Ryan

Reviewer Comments to Author:

This paper describes a high-quality genome assembly of Portunus trituberculatus, one of the most widely fished species of crab in the world. The paper is written clearly and succinctly. The figures look excellent are clear and informative. I downloaded the genome and found an expected Hox cluster, which is in line with this being a high quality data set. The authors do not describe the Hox cluster and this is fine, but they should consider since it would not have to be that extensive of an analysis and a description plus Hox complex figure would increase the interest in the paper (it could also be a follow-up study). Nevertheless, this is a wonderful genomic resource, an excellent analysis, and if the authors address the reproducibility issues in my next paragraph, I would say that this is a model genome data note.

The methods appear thorough, however repeating these analyses in full would be impossible without guessing at some parameter settings etc. In order to make the work repeatable, please include ALL command lines in a supplemental document. There is an excellent example in the supplement linked here:
https://academic.oup.com/mbe/article/35/2/486/4644721#113627427

Line 34: "only limited transcriptome data currently available"
--This is untrue as there is a draft genome assembly available in GenBank:
https://www.ncbi.nlm.nih.gov/nuccore/VSRR000000000.1
This available draft should be acknowledged in the manuscript (even though the assembly in this current study is far superior).

Line 70: "genomic research on the swimming crab has only been conducted at the transcriptome level[14-16], with the whole genome not yet described.
--Likewise, this line should be updated to mention this draft genome.

Line 84: "Muscle RNA was also extracted using TRIzol (Invitrogen) according to the manufacturer's instructions"
-- Should clarify whether the same animal used was used for extraction of RNA asthe genome. Indeed it should also be noted if the same animal was used for all genomic sequencing.

Line 297 (and line 300): "greater survival pressures on these two species"
--I wouldn't attribute faster evolutionary rates to "survival pressures." Evolutionary rate has more to do with generation time (shorter=greater) and population size (larger=greater). The evolutionary rate makes sense in relation to both of these factors. Differences in survival pressures are heavily influenced by competition in large populations.

Table 2: It seems as if the "Summary" row represents the percentage for "Complete BUSCO (C)." The

label "Summary" does not make sense in this context. I would rename to "Summary (percentage Complete Busco)" or "percentage Complete Busco"
I love Figure 1! Beautiful creature!
Figure 2 and 3 legends should include more information. For example, what program was used to generate figure. What is the underlying data from, etc.

Level of Interest

Please indicate how interesting you found the manuscript: Choose an item.

Quality of Written English

Please indicate the quality of language in the manuscript: Choose an item.

Declaration of Competing Interests

Please complete a declaration of competing interests, considering the following questions:

- Have you in the past five years received reimbursements, fees, funding, or salary from an organisation that may in any way gain or lose financially from the publication of this manuscript, either now or in the future?
- Do you hold any stocks or shares in an organisation that may in any way gain or lose financially from the publication of this manuscript, either now or in the future?
- Do you hold or are you currently applying for any patents relating to the content of the manuscript?
- Have you received reimbursements, fees, funding, or salary from an organization that holds or has applied for patents relating to the content of the manuscript?
- Do you have any other financial competing interests?
- Do you have any non-financial competing interests in relation to this paper?

If you can answer no to all of the above, write 'I declare that I have no competing interests' below. If your reply is yes to any, please give details below.

I declare that I have no competing interests

I agree to the open peer review policy of the journal. I understand that my name will be included on my report to the authors and, if the manuscript is accepted for publication, my named report including any attachments I upload will be posted on the website along with the authors' responses. I agree for my report to be made available under an Open Access Creative Commons CC-BY license (http://creativecommons.org/licenses/by/4.0/). I understand that any comments which I do not wish to

be included in my named report can be included as confidential comments to the editors, which will not be published.

Choose an item.

To further support our reviewers, we have joined with Publons, where you can gain additional credit to further highlight your hard work (see: https://publons.com/journal/530/gigascience). On publication of this paper, your review will be automatically added to Publons, you can then choose whether or not to claim your Publons credit. I understand this statement.

Yes Choose an item.
